# Systematic review on the impact of the pneumococcal conjugate vaccine ten valent (PCV10) or thirteen valent (PCV13) on all-cause, radiologically confirmed and severe pneumonia hospitalisation rates and pneumonia mortality in children 0-9 years old

**DOI:** 10.7189/jogh.13.05002

**Published:** 2023-02-03

**Authors:** Rita Reyburn, Anthea Tsatsaronis, Claire von Mollendorf, Kim Mulholland, Fiona M Russell, ARI Review group, Trevor Duke, Hamish Graham, Steve Graham, Amy Gray, Amanda Gwee, Claire von Mollendorf, Kim Mulholland, Fiona Russell, Maeve Hume-Nixon, Saniya Kazi, Priya Kevat, Eleanor Neal, Cattram Nguyen, Alicia Quach, Rita Reyburn, Kathleen Ryan, Patrick Walker, Chris Wilkes, Poh Chua, Yasir Bin Nisar, Jonathon Simon, Wilson Were

**Affiliations:** 1 Murdoch Children’s Research Institute, Melbourne, Victoria, Australia; 2 Faculty of Medicine, Dentistry and Health Sciences, The University of Melbourne, Melbourne, Victoria, Australia; 3 Department of Paediatrics, The University of Melbourne, Melbourne, Victoria, Australia; 4 London School of Hygiene and Tropical Medicine, London, UK

## Abstract

**Background:**

There is an ongoing need to assess the impact of pneumococcal conjugate vaccines (PCVs) to guide the use of these potentially valuable but under-utilized vaccines against pneumonia, which is one of the most common causes of post-neonatal mortality.

**Methods:**

We conducted a systematic review of the literature on PCV10 and PCV13 impact on all-cause, radiologically confirmed and severe pneumonia hospitalisation rates as well as all-cause and pneumonia-specific mortality rates. We included studies that were published from 2003 onwards, had a post-licensure observational study design, and reported on any of our defined outcomes in children aged between 0-9 years. We derived incidence rates (IRs), incidence rate ratios (IRRs) or percent differences (%). We assessed all studies for risk of bias using the Effective Public Health Practice Project (EPHPP) quality assessment tool.

**Results:**

We identified a total of 1885 studies and included 43 comparing one or more of the following hospitalised outcomes of interest: all-cause pneumonia (n = 27), severe pneumonia (n = 6), all-cause empyema (n = 8), radiologically confirmed pneumonia (n = 8), pneumococcal pneumonia (n = 7), and pneumonia mortality (n = 10). No studies evaluated all-cause mortality. Studies were conducted in all WHO regions except South East Asia Region (SEAR) and low- or middle-income countries (LMICs) in the Western Pacific Region (WPR). Among children <5 years old, PCV impact ranged from 7% to 60% for all-cause pneumonia hospitalisation, 8% to 90% for severe pneumonia hospitalisation, 12% to 79% for radiologically confirmed pneumonia, and 45% to 85% for pneumococcal confirmed pneumonia. For pneumonia-related mortality, impact was found in three studies and ranged from 10% to 78%. No obvious differences were found in vaccine impact between PCV10 and PCV13. One study found a 17% reduction in all-cause pneumonia among children aged 5-9 years, while another found a reduction of 81% among those aged 5-17 years. A third study found a 57% reduction in all-cause empyema among children 5-14 years of age.

**Conclusion:**

We found clear evidence of declines in hospitalisation rates due to all-cause, severe, radiologically confirmed, and bacteraemic pneumococcal pneumonia in children aged <5 years, supporting ongoing use of PCV10 and PCV13. However, there were few studies from countries with the highest <5-year mortality and no studies from SEAR and LMICs in the WPR. Standardising methods of future PCV impact studies is recommended.

Pneumonia is the single largest infectious cause of childhood mortality globally, accounting for 14% of deaths in children <5 years old [1]. The burden of disease is predominantly present in children <2 years old and in South East Asian and African regions [2]. The bacterium *Streptococcus pneumoniae*, known as the pneumococcus, is responsible for a significant proportion of this global burden and is estimated to cause 18.3% of severe bacterial pneumonia and 30% of all pneumonia deaths in childhood [2].

Pneumococcal conjugate vaccines (PCVs) have been in use for more than 20 years with increasing global coverage. By the end of 2019, PCVs had been introduced in 149/195 (76%) countries and global coverage was estimated to be 48% [3]. However, cost has been a major barrier to introduction, especially for middle-income countries. Currently three PCVs are WHO-pre-qualified. Two are 10-valent PCV10s (*Synflorix* and *Pneumosil*), covering different serotypes, and one is 13-valent PCV13 (*Prevnar13*®). The recently WHO-pre-qualified PCV10, *Pneumosil*, is less expensive. The WHO recommends a three-dose schedule delivered either as a two dose primary series with one booster (2 + 1) or a three dose primary series (3 + 0) [4].

Studies evaluating the impact of PCV10 and PCV13 have been conducted in various settings. PCV10 and PCV13 have been found to be effective at reducing hospitalisation for invasive pneumococcal disease (IPD), clinical pneumonia, and radiologically confirmed pneumonia both directly (in age groups targeted for vaccination) and indirectly (in unvaccinated individuals living in close proximity to other vaccinated individuals) when used in a three dose (3 + 0 or 2 + 1) or four dose (3 + 1) schedule [4,5]. These studies face several challenges, most particularly the absence of a gold standard case definition of pneumonia. For pneumococcal pneumonia, the causative organism is often not isolated and radiologically confirmed, and clinical features vary widely [6]. Additionally, there are constraints in the quality or availability of radiologically confirmed diagnosis and functioning health information systems in resource-poor countries that bear the highest burden of disease. Therefore, for epidemiological purposes, there are numerous pneumonia outcomes that are commonly used to measure the impact of PCV: hospitalised all-cause pneumonia, severe pneumonia (2005 or 2013 WHO definition), radiologically confirmed pneumonia, and bacteraemic pneumococcal pneumonia or mortality. There is limited data on the indirect impact of PCV10 and PCV13 on pneumonia burden in children aged above five years, as identified in our literature search.

We aimed to summarize the impact of the PCVs used in national immunisation programs (NIPs) for three years or more, in children 0-9 years old with hospitalised pneumonia using the following five outcomes: all-cause pneumonia hospitalisation, radiologically confirmed pneumonia hospitalisation, severe pneumonia (including empyema) hospitalisation, pneumonia mortality, and all-cause mortality.

## METHODS

### Literature search

We searched Medline (Ovid), Embase (Ovid), and Cochrane Library databases using Medical Subject Headings (MeSH), thesaurus terms, and keywords to identify relevant studies. The search strategy and keywords are available in the **Online Supplementary Document**. We additionally searched PubMed using keywords only, to source any publications and items not indexed in Medline. We manually searched the references of selected relevant published systematic reviews to ensure that important studies were not missed in the initial search. We then assessed the compiled publications, with two reviewers first screening their titles and abstracts followed by their full text. Disagreements on the inclusion were resolved through discussion among the reviewers.

### Inclusion and exclusion criteria

We limited the database searches to studies published from 2003 onwards, as the first PCV was introduced globally in 2000 and one of our inclusion criteria was its use at least for three years or more post-introduction. We further applied limits to exclude randomized controlled trials, case-control studies, case reports, and case series studies, as the impact of a vaccination programs is evaluated with post-licensure observational studies [7,8]. We also restricted the searches to English language publications. The inclusion criteria for this review were: post-licensure observational studies reporting on incidence rates, rate ratios, or percent difference and comparing the outcomes of hospitalised pneumonia, all-cause empyema, all-cause mortality, or pneumonia mortality in any time period before to at least three years after the introduction of either PCV10 or PCV13 into the NIP, allowing more time for indirect and total effects to occur [9]. Full details of the inclusion and exclusion criteria are available in the **Online Supplementary Document**. We include studies in settings where PCV10 or PCV13 were the first PCV to be introduced into the NIP or where PCV7 was first introduced and subsequently replaced by either PCV10 or PCV13. We excluded studies if the population did not include children 0-9 years, if less than 50% of the catchment population received PCV in the post-PCV period, or if they compared only the PCV7 period to PCV10 or PCV13 period (Table S1 in the **Online Supplementary Document**). Two reviewers independently extracted the data and any disagreements were resolved through discussion.

We used the Effective Public Health Practice Project (EPHPP) quality assessment tool for quantitative studies [10] to assess the level of risk of bias for each study, as well as to evaluate their internal validity of each study was evaluated considering eight methodological – selection bias of study participants, study design, confounders, blinding, data collection methods, withdrawals of study participants, intervention integrity, and analysis. The protocol is available upon request from the authors.

We did not conduct meta-analyses due to the heterogeneity between studies and case definitions of pneumonia outcomes (case definitions are shown in Table S2 in the **Online Supplementary Document**). Study results were stratified by age group, world bank country income status, time since PCV introduction, and PCV valency.

## RESULTS

**Figure 1:**
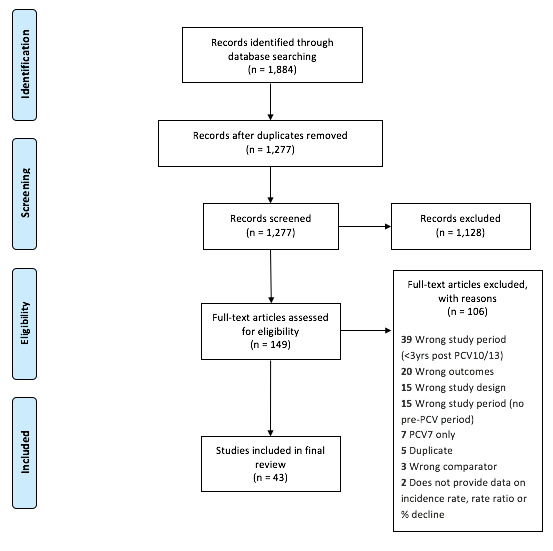
Flow diagram for study selection.

We identified a total of 1885 articles, 636 in Embase, 547 in Medline (Ovid), 410 in PubMed, and 291 in Cochrane Library. We did not find any additional missed papers through the manual examination of the references of selected relevant published systematic reviews. After excluding 608 duplicates, a total of 1277 studies were screened by title and abstract; 149 studies were subsequently screened in the full text review, with 43 studies included in the final review (**Figure 1**). The results of the EPHPP quality assessment are shown in the **Online Supplementary Document**.

Of the 43 studies included, 27 evaluated hospitalisation due to all-cause pneumonia, 6 severe pneumonia, 8 all-cause empyema, 8 radiologically confirmed pneumonia, 7 pneumococcal pneumonia and 10 pneumonia mortality. No studies directly evaluated all-cause mortality (Table 1). It is important to note the wide variety of case definitions for each outcome (Table 1). Twenty-one (43%) studies looked at high-income countries (HIC), 17 (35%) at upper-middle income countries (UMIC), 7 (14%) at lower-middle income countries (LMICs) and 4 (8%) at low-income countries (LIC). Studies were from all WHO regions apart from SEAR or LMICs in WPR. Out of the 43 studies, 23 (53%) included children aged <5 years only, while 20 (47%) included children 0-9 years of age. Twenty-one (49%) studies examined PCV13, 16 (37%) PCV10 and 6 (14%) both PCV10 and PCV13 combined.

**Table 1:** PCV impact by age group, outcome and country income status

Outcome	Income status	<2y			2-4y			<5y			5-9y			<18y		
		**Range of decline in point estimate**	**No. of study results that changed in the post-PCV period**	**No. of study results that did not change in the post-PCV period**	**Range of decline in point estimate**	**No. of study results that changed in the post-PCV period**	**No. of study results that did not change in the post-PCV period**	**Range of decline in point estimate**	**No. of study results that changed in the post-PCV period**	**No. of study results that did not change in the post-PCV period**	**Range of decline in point estimate**	**No. of study results that changed in the post-PCV period**	**No. of study results that did not change in the post-PCV period**	**Range of decline in point estimate**	**No. of study results that changed in the post-PCV period**	**No. of study results that did not change in the post-PCV period**
**All-cause pneumonia**	**LIC**	Decline but no point estimate	1	0	Decline but no point estimate	1	0	9%-22%	4	0	-	0	0	-	0	0
	**LMIC**	8%-38%	5	1	5%	1	0	29%-35%	2	1	No change	1	0	-	0	0
	**UMIC**	13%-39%	7	0	7%-22%	3	0	8%-39%	3	0	17%	1	0	-	0	0
	**HIC**	7%-60%	13	1	20%-47%	6	0	8%-18%	2	1	12%-81%	6	1	43%-40%	2	0
**Severe pneumonia**	**LIC**	Decline but no point estimate	1	0	Decline but no point estimate	1	0	Decline but no point estimate	1	0	-	0	0	-	0	1
	**LMIC**	-	0	0	-	0	0	-	0	0	-	0	0	-	0	0
	**UMIC**	-	0	0	-	0	0	-	0	0	-	0	0	-	0	0
	**HIC**	51%	1	0	30%	1	0	90%	1	0	10%-58%	2	1	-	0	0
**All-cause empyema**	**LIC**	-	0	0	-	0	0	-	1	3	48%-57%	2	2	-2%-56%	2	1
	**LMIC**	-	0	0	-	0	0	-	0	0	-	0	0	-	0	0
	**UMIC**	-	0	0	-	0	0	-	0	0	-	0	0	-	0	0
	**HIC**	33%-68%	2	1	42%-43%	1	1	-	0	0	57%	1	0	-	0	0
**Radiologically confirmed pneumonia**	**LIC**	23%-29%	2	0	22%	1	0	-	0	0	-	0	0	-	0	0
	**LMIC**	-	0	2	-	0	1	48%	1	0	-	0	1	-	0	0
	**UMIC**	25%-70%	5	0	12%-73%	3	0	65%	1	0	-	0	0	-	0	0
	**HIC**	53%-57%	2	2	60%-79%	2	1	40%-70%	4	0	41%	1	1	-	0	0
**Pneumococcal Pneumonia**	**LIC**	58%-86%	4	0	57%-66%	2	0	-	0	0	-	0	0	-	0	0
	**LMIC**	-	0	0	-	0	0	-	0	0	-	0	0	-	0	0
	**UMIC**	-	0	0	-	0	0	-	0	0	-	0	0	-	0	0
	**HIC**	52%-81%	5	0	53%-77%	2	0	45%-70%	5	0	69%	1	0	-	0	0
**Pneumonia mortality**	**LIC**	Decline but no point estimate	1	0	Decline but no point estimate	1	0	Decline but no point estimate	1	0	-	0	0	-	0	0
	**LMIC**	-	0	1	-	0	0	-	0	2	-	0	1	-	0	0
	**UMIC**	2%-40%	12	2	1%-52%	5	2	2%-78%	7	4	-	0	0	-	0	0
	**HIC**	-	0	0	-	0	0	-	0	1	-	0	1	18%	1	0

PCV – pneumococcal vaccine, y – years, LIC – low-income country, LMIC – low- and middle-income country, UMIC – upper-middle income country, HIC – high-income country

### All-cause pneumonia hospitalisation

Twenty-seven of the 43 included studies evaluated the effect of PCV10 or PCV13 on all-cause pneumonia hospitalisations [11-37] (Table S3 in the **Online Supplementary Document**). When we considered the 22 studies reporting vaccine impact with either an incidence rate ration (IRR) or percent decline and included age groups which were below 10 years of age [11-17,20,21,23,24,26-31,33-37], the median decline in all-cause pneumonia hospitalisation was 21% and ranged between 4% to 64% [29] Study results by age group were: for age group <12 months, median = 28% (range = 4% [23] to 55% [24]); for age group 12-23 months, median = 21% (range = 8% [15] to 48% [34]); for age group 24-59 months median = 22% (range = 7% [33] to 23% [12]); for age group <5 years, median = 18% (range = 7% [16] to 60% [37]); for age group 5-9 years one study found a reduction of 17% [13] and or children aged 5-17 years, median = 49% (range = 16% [29] to 81% [27]).

Three studies used time series analysis. Among Botswanan children aged <5 years there was a 6% annual decline [19]. Among Brazilian children aged <2 years annual declines were 4%, among children aged 2-4 years annual declines were 11% and among children aged 5-9 years there was no change [25]. Among children aged <5 years in Burkina Faso there was a 0.66 (95% confidence interval (CI) = 0.51, 0.84) change in intercept and a 0.97 (95% CI = 0.96, 0.98) change in slope, with sub-analysis by age groups; 0-23 months showed a 0.76 (95% CI = 0.59, 0.98) change in intercept and a 0.96 (95% CI = 0.95, 0.97) change in slope and 24-59 months showed a 0.50 (95% CI = 0.36, 0.70) change in intercept and a 0.98 (95% CI = 0.97, 1.002) change in slope [38]. Eight studies showed no change in one of the age groups reviewed.

### Severe pneumonia hospitalisations

Six included studies evaluated the effect of PCV10 or PCV13 on severe pneumonia hospitalisations [35,38-42] (Table S4 in the **Online Supplementary Document**). One study claimed to use the WHO 2005 definition of severe pneumonia, while five studies did not state which version was used. When we considered the four studies reporting PCV10 and PCV13 impact as an IRR and included age groups below 10 years of age [35,40-42], the median decline in severe pneumonia hospitalisation was 52% and ranged between 8% [40] to 90% [42]. Study results by age group were: for age group <12 months, median = 54% (range = 51% [35] to 57% [40]); for age group 12-23 months, median = 56 (range = 39% [43] to 72% [40]; for age group 24-59 months, median = 41% (range = 30% [35] to 56% [12,44]); for age group <5 years, median = 58% (range = 8% [40] to 90% [42]); and no studies reported vaccine impact for age groups 5-9 years. One study used a time series analysis. Among children aged <5 years in Burkina Faso, there was a 0.64 (95% CI = 0.49, 0.84) change in intercept and a 0.970 (95% CI = 0.96, 0.98) change in slope, with sub-analysis by age groups; 0-23 months showed a 0.74 (95% CI = 0.56; 0.98) change in intercept and a 0.959 (95% CI = 0.944; 0.974) change in slope and 24-59 months showed a 0.45 (95% CI = 0.30, 0.68) change in intercept and a 0.996 (95% CI = 0.97, 1.02) change in slope [38].

Four studies [40] included results which showed no change, with only one showing no change in all outcomes and age groups; this study conducted in Canada assessed changes in the specific case definition of pediatric complicated pneumonia (empyema, parapneumonic effusion, necrotizing pneumonia, and lung abscess) among children <18 years and found that serotype replacement following PVC7 had contributed to increased rates of empyema [39]. The other three studies [40] all used multiple age groups and outcomes and found a proportion of their point estimates did not indicate a decline. A study in Kenya assessing two different severe pneumonia outcomes in five age groups found that all 10-point estimates declined, but that the the confidence intervals (CIs) for 7/10 results crossed the null [43]. One study in the Netherlands showed a decline in the point estimate, but the CIs crossed the null for the age group 5-17 years, and a 90% decline was observed among children <5 years [42]. A study from The Gambia found no decline in clinical pneumonia among 1-4-year-olds, but a large reduction in hypoxic pneumonia among the same population and age group [40].

### All-cause empyema hospitalisations

Eight included studies evaluated the PCV10 or PCV13 on all-cause empyema hospitalisations [12,30,32,35,42,45-47], a sub-category of severe pneumonia (Table S5 in the **Online Supplementary Document**). All studies focused on the age group <5 years and showed a median decline of 43%, ranging from 23% [46] to 68% [46]. The minimum and maximum declines were from the same study and age group (<2 years of age), but the outcome differed; parapneumonic empyema reduced by 23%, while the more specific outcome of pneumococcal parapneumonic empyema reduced by 68%. No studies assessed vaccine impact against all-cause empyema hospitalisation among children <12 months, 12-23 months, 24-59 months, or 5-9 years of age separately. One study in the UK reported a 57% decline in the rate of hospitalisation among children aged 5-14 years with empyema [35].

### Radiologically confirmed pneumonia hospitalisations

Eight included studies evaluated the PCV10 or PCV13 on radiologically confirmed pneumonia hospitalisations [16,33,40,41,48-51] (Table S6 in the **Online Supplementary Document**). All but two [48,50] specified that they used the WHO-defined radiologically confirmed pneumonia definition [52]. When we considered these eight studies, which included age groups below 10 years of age, the median decline in radiologically confirmed pneumonia hospitalisation was 38% (range = 12% [33] to 79% [51]). Study results by age group were: for age group <12 months, median = 50% (range = 23% [40] to 53% [49]); for age group 12-23 months, median = 29% (range = 25% [33] to 70% [49]); for age group 24-59 months, median = 60% (range = 12% [33] to 79% [51]); for age group <5 years, median = 48% (range = 22% [40] to 70% [51]); and there were no vaccine impact studies for the 5-9 years age group. Four studies among age groups <12 months, 12-23 months, 24-59 months, 5-10 years, and 5-14 years showed no change.

### Pneumococcal pneumonia hospitalisations

Seven included studies evaluated the impact of PCV10 or PCV13 on pneumococcal pneumonia hospitalisations [30,35,40,50,53-55] (Table S7 in the **Online Supplementary Document**). When we considered the five studies reporting PCV10 and PCV13 impact as an IRR and included age groups below 10 years of age [35,40,53-55], the median decline in pneumococcal pneumonia hospitalisation was 67% (range = 45% [55] to 85% [54]). Study results by age group were: for age group <12 months, median = 62% (range = 52% [53] to 66% [55]); for age group 12-23 months, median = 75% (range = 67% [53] to 75% [40]); for age group 24-59 months, median = 64% (range = 53% [35] to 77% [53]); for age group <5 years, median = 69% (range = 45% [55] to 85% [54]); and no studies assessed vaccine impact for age groups 5-9 years. A study from Finland showed a relative rate reduction of 77 (95%CI = 64-86) among age groups 3-24 months and 70 (95% CI = 49, 84) among age groups 7-71 months [30]. A study from Japan reported a decline but no point estimate [50].

### Pneumonia-related mortality

Ten included studies evaluated the PCV10 or PCV13 on pneumonia mortality [15,18,19,25,28,29,33,38,42,56] (Table S8 in the **Online Supplementary Document**), three of which showed an impact on mortality. Among children aged <5 years in Burkina Faso, there was a 0.49 (95% CI = 0.31, 0.78) change in intercept and a 0.97 (95% CI = 0.94, 0.99) change in slope. When further sub-analyzed by age groups, the following was observed; the 0-23 months age group showed a 0.60 (95% CI = 0.37, 0.99) change in intercept and a 0.95 (95% CI = 0.92, 0.97) change in slope and 24-59 months showed a 0.22 (95% CI = 0.08, 0.60) change in intercept and a 1.03 (95% CI = 0.98, 1.08) change in slope [38]. Among Spanish children <18 years in-hospital mortality fell by an odds ratio of 0.82 (95% CI = 0.77, 0.89) [25]. Among Botswanan children aged 1-59 months there was a 22% (95% CI = 8, 33) annual decline in the five years post PCV13 introduction [19].

The seven remaining studies showed no change in mortality: among Nicaraguan children aged 0-4 years, the IRR = 1.01 (95% CI = 0.67, 1.52) and 5-14 years IRR = 0.22 (95% CI = 0.02, 2.47) [15]. Among Dutch children aged <5 years, the IRR was 1.46 (95% CI = 0.37, 5.80); among those aged 5-17 years, the IRR was 0.88 (95% CI = 0.06, 13.3) [42]. Among Swedish children aged <2 years, the IRR was 0.36 (95% CI = 0.07, 1.83), while it was 0.42 (95% CI = 0.08, 2.31) among those aged 2-4 years and 0.9 (95% CI = 0.35, 2.33) among those aged 5-17 years [29]. Three studies were conducted in Brazil and all noted dramatic declines in mortality in the pre-PCV era. One of these studies showed no decline in pneumonia mortality associated with PCV introduction among children aged <5 years using their primary analysis synthetic control time series, but the secondary time trend analysis showed an approximately 10% decline in mortality, which was greater (in areas in lower socio economic status 20%-50%, depending on the age group) [56]. The second study in Brazil showed an annual decline of 6.5% (95% CI = 6.3, 6.7) in the post-PCV period among children <5 years old, but this was not statistically significant [18]. The third Brazilian study among children <5 years showed no decline in the case-fatality ratio [33]. All three Brazilian studies noted a substantial decline in child mortality in the years prior to PCV introduction. Among Zambian children, there was no change in in-hospital deaths among children <5 years (no point estimate for the change was provided) [28].

### PCV impact by country income status, vaccine valency, and time since introduction

PCV impact on pneumonia hospitalisation appears to be slightly higher in high-income countries (HICs) compared to low-income countries (LIC), LMICs, or upper-middle income countries (UMICs), but the heterogeneity in study results make it challenging to draw strong conclusions (**Table 1**). PCV impact on pneumonia was similar for PCV10 and PCV13 (**Table 2**). There is some evidence that PCV impact on pneumonia was slightly higher in the studies which were conducted >5 years vs 3-5 years post-PCV introduction. However, there were few studies in the former group, so definitive conclusions cannot be drawn (**Table 3**).

**Table 2:** PCV impact by age group, outcome and PCV valency

Outcome	PCV valency	<2y			2-4y			<5y			5-9y			<18y		
		**Range of decline in point estimate**	**No. of point estimates which changed in post-PCV period**	**No. of point estimates which did not change in post-PCV period**	**Range of decline in point estimate**	**No. of point estimates which changed in post-PCV period**	**No. of point estimates which did not change in post-PCV period**	**Range of decline in point estimate**	**No. of point estimates which changed in post-PCV period**	**No. of point estimates which did not change in post-PCV period**	**Range of decline in point estimate**	**No. of point estimates which changed in post-PCV period**	**No. of point estimates which did not change in post-PCV period**	**Range of decline in point estimate**	**No. of point estimates which changed in post-PCV period**	**No. of point estimates which did not change in post-PCV period**
**All-cause pneumonia**	**PCV10**	13%-48%	13	2	7%-22%	4	0	8%-29%	4	2	12%-26%	3	0	-	0	0
	**PCV13**	8%-60%	10	0	5%-44%	5	1	9%-39%	6	0	33%-81%	3	1	43%-49%	2	0
**Severe pneumonia**	**PCV10**	49%	1	1	61%	1	0	40%-90%	2	0	10%-17%	2	0	-	0	0
	**PCV13**	2%-72%	5	1	30%-56%	3	1	8%-61%	4	0	58%	1	1	-	0	0
**All-cause empyema**	**PCV10**	-	0	1	-	0	2	-	0	1	-	0	0	-	0	0
	**PCV13**	33%-68%	4	0	42%-43%	2	0	-	0	1	48%-57%	2	1	-2%-56%	2	1
**Radiologically confirmed pneumonia**	**PCV10**	25%	3	4	12%-23%	2	2	40%-48%	0	2	-	0	1	-	0	0
	**PCV13**	23%-70%	6	0	22%-79%	4	0	45%-70%	4	0	41%	1	0	-	0	0
**Pneumococcal Pneumonia**	**PCV10**	77%	1	0	-	0	0	70%-85%	2	0	-	0	0	-	0	0
	**PCV13**	52%-86%	8	0	53%-77%	4	0	45%-67%	4	0	69%	1	0	-	0	0
**Pneumonia Mortality**	**PCV10**	3%-27%	6	3	28%-52%	2	2	24%-25%	2	6	-	0	1	-	0	0
	**PCV13**	2%-35%	7	0	1%-10%	4	0	2%-78%	6	1	-	0	1	-	0	0

PCV – pneumococcal vaccine, y – years

**Table 3:** PCV impact by age group, outcome and time since PCV introduction

Outcome	Years since PCV introduction	<2			2-4y			<5y			5-9y			<18y		
		**Range of decline in point estimate**	**No. of point estimates which changed in post-PCV period**	**No. of point estimates which did not change in post-PCV period**	**Range of decline in point estimate**	**No. of point estimates which changed in post-PCV period**	**No. of point estimates which did not change in post-PCV period**	**Range of decline in point estimate**	**No. of point estimates which changed in post-PCV period**	**No. of point estimates which did not change in post-PCV period**	**Range of decline in point estimate**	**No. of point estimates which changed in post-PCV period**	**No. of point estimates which did not change in post-PCV period**	**Range of decline in point estimate**	**No. of point estimates which changed in post-PCV period**	**No. of point estimates which did not change in post-PCV period**
**All-cause pneumonia**	**3-5y**	2%-45%	23	2	5%-47%	11	1	8%-35%	9	2	12%-81%	7	2	43%-49%	2	0
	**>5y**	20%-48%	3	0	-	0	0	39%	2	0	-	0	0	-	0	0
**Severe pneumonia**	**3-5y**	2%-72%	6	2	30%-61%	4	1	8%-90%	6	0	10%-58%	3	1	-	0	1
	**>5y**	-	0	0	-	0	0	-	0	0	-	0	0	-	0	0
**All-cause empyema**	**3-5y**	33-68	4	1	42%-43%	2	1	-	0	3	48%-57%	2	2	-	0	1
	**>5y**	-	0	0	-	0	0	-	0	0	-	0	0	-2%-65%	2	0
**Radiologically confirmed pneumonia**	**3-5y**	-	0	2	-	0	1	40%-45%	2	0	41%	1	0	-	0	0
	**>5y**	53%-57%	2	0	60%-79%	2	0	55%-70%	2	0	-	0	0	-	0	0
**Pneumococcal pneumonia**	**3-5y**	66%-81%	3	0	53%	1	0	54%-70%	4	0	69%	1	0	-	0	0
	**>5y**	52%-67%	2	0	77%	1	0	67%	1	0	-	0	0	-	0	0
**Pneumonia mortality**	**3-5y**	2%-40%	9	3	1%-52%	5	2	2%-25%	6	5	1	0	2	18%	1	0
	**>5y**	3%-27%	4	0	28%	1	0	11%-78%	2	2	-	0	0	-	0	0

PCV – pneumococcal vaccine, y – years

## DISCUSSION

Our findings reinforce the current global evidence of the effectiveness of PCV10 and PCV13 against pneumonia and support their continued and extended use in all settings. We found a consistent decline in pneumonia hospitalisations incidence among both vaccine eligible and non-vaccine eligible children despite the considerable heterogeneity in the magnitude of the decline, which is broadly consistent with the findings of the PCV Review of Impact Evidence (PRIME), an extensive WHO review of the impact of PCVs undertaken in 2017 [8]. Our review, to some extent, is an update of PRIME and includes higher-valency PCVs and a longer observation period since vaccine introduction. Reduction in mortality due to pneumonia-specific causes was evident in some, but not all studies. However, there was a lack of PCV impact studies undertaken in settings with the highest under-five mortality.

All WHO regions were represented, apart from SEAR and low- or middle-income countries in WPR. However, a recently published study from Fiji found a 21%-46% reduction in hospitalised pneumonia among children 2-23 months and 2-4 years of age, depending on the case definition and age group [57]. Few countries in SEAR and WPR have introduced PCV. Of the 27 countries in WPR, only 67% (n = 18) have PCV in their NIP [58]. A study published after our search date from Bangladesh showed that PCV10 was 47% (95% CI = 11, 69) effective against radiographically confirmed pneumonia among children aged 3-11 months of age [59]. A phased introduction evaluation of PCV in Mongolia is ongoing, but preliminary findings have found a 24%-63% reduction in incidence pneumonia rates (as of December 2019), depending on which pneumonia outcome was used (ranging from all to very severe pneumonia) in children 2-59 months of age (Claire von Mollendorf, personal communication, 2021).

Our review has demonstrated the impact of PCV on reducing pneumonia incidence and mortality across all included age groups, particularly evident for three outcomes: all-cause hospitalization, radiologically confirmed, and pneumococcal pneumonia. There was a trend of rising impact as the pneumonia outcome increased in specificity, as exemplified by children <5 years of age showing the highest declines for bacteraemic pneumococcal pneumonia, then followed by radiologically confirmed pneumonia, the historical gold standard epidemiological outcome used in clinical trials [60-63]. Rates of hospitalised pneumonia are not a good indicator of trends in pneumonia incidence, as changes in admission criteria and referral patterns may lead to apparent increases or decreases in hospitalised pneumonia, even in the absence of any intervention.

We would expect the decline to be greatest in infants, as they experience the highest incidence of WHO-defined pneumonia and are the target group for vaccination, although the relative impact of PCV may be affected with increasing incidence of respiratory syncytial virus (RSV) [64]. This was demonstrated in a previous systematic review that found higher cumulative reductions in children <2 years compared to children aged 24-59 months of age for both clinical and radiologically confirmed pneumonia [65]. However, we did not detect a trend of decreasing impact with increasing age, most probably due to the heterogeneity in case definitions and settings across studies. Children <2 years of age are most affected by pneumococcal pneumonia; however, the vaccine covers only 10-13 pneumococcal serotypes that cause most invasive pneumococcal disease. It is unclear if the introduction of PCV has shifted the age distribution of pneumonia cases, and/or caused increases in replacement disease, so these should be assessed further.

Only three studies [13,27,35] included the older pediatric population (children ≥5 years), two assessed the impact on all-cause pneumonia hospitalisation [13,27] and the other on all-cause empyema hospitalisation [35], and all studies documented a decline in their respective outcomes following PCV introduction. All studies were from high-income settings. Further data on this age group from low- and middle-income settings is needed. The impact of serotype replacement on pneumonia outcomes is unknown. Serotype 3, generally considered a vaccine failure, is a common cause of pneumococcal empyema in the post-PCV era [66,67]. Reductions were found in the few studies from HICs assessing PCV impact on empyema hospitalisation incidence. However, as serotype replacement may become more apparent over time and may differ by the degree of pneumococcal transmission, the etiology of empyema and bacteraemic pneumonia should be monitored along with other invasive pneumococcal disease, especially pneumococcal meningitis.

It is important to recognize that 54% of the global burden of pneumonia mortality is concentrated in five countries (India, Nigeria, Pakistan, Democratic Republic of the Congo, and Ethiopia) [68], all of which introduced PCV at some point between 2011 and 2017, but none are included in any of the PCV impact studies we reviewed. Three studies from Burkina Faso [38], Botswana [19], and Spain [25] demonstrated a decline in pneumonia mortality and an additional four studies demonstrated a modest decline in pneumonia mortality, but 95% confidence intervals crossed the null [15,29,42,56], possibly due to small case numbers. Mortality is a rare outcome in many countries where impact studies were conducted and all studies are hospital based and would therefore not capture deaths which occur outside of the hospital setting. A recent study from Fiji found mortality reduced by 39% for all-cause pneumonia/bronchiolitis/asthma admissions in children 2-23 months old [57]. In high mortality settings, there is a need for large-scale studies to demonstrate the evidence of vaccine impact on mortality [69].

We found that the impact on pneumonia was similar for PCV10 and PCV13. The only head-to-head clinical trial comparison of PCV10 and PCV13 found similarly highly immunogenicity for individual serotypes when used in 2 + 1 schedule, which is expected to translate into similar vaccine impact [70].

There is some suggestion that PCV impact on pneumonia was slightly higher in studies that collected post-PCV data for more than five years compared to 3-5 years. However, there were few studies in the former group, making it difficult to draw conclusions. A longer time from vaccine introduction would expectedly elicit greater population impact as PCV prevents carriage of vaccine serotype pneumococci with an expected greater impact on unvaccinated age groups over time. Additionally, other relevant factors such as variations in case definition and settings could not be accounted for in the cross tabulation used for the sub-group analysis.

To improve pneumococcal detection and serotyping capacity, countries, especially those with high mortality in children <5 years should be supported to join the Tier 1, 2, and 3 (invasive disease, including meningitis and pneumonia) of the WHO Invasive Bacterial Vaccine Preventable Diseases Surveillance global network to monitor trends in meningitis and severe pneumonia admissions, empyema and deaths, which includes serotyping of invasive samples. Sentinel sites should also include hospitals in high-risk areas. Similarly, PCV may have changed the age distribution of pneumococcal disease. There is a need to understand the age distribution of pneumococcal pneumonia cases following the introduction of PCV.

### Limitations

The main limitation of this review is the heterogeneity in the pneumonia case definitions and analytical methods prohibiting a meta-analysis. Although studies agreed on the radiologically confirmed and culture positive pneumococcal pneumonia definitions, defining clinical features varied across studies according to the locally available clinical data. This heterogeneity would influence the sensitivity or specificity in detecting pneumococcal disease [71], preventing the comparison of results across studies [60]. The 2013 revision of the 2005 WHO definition of severe pneumonia regarding admission criteria affects the apparent incidence rates and therefore may affect the findings of PCV impact studies [25]. Most studies did not document whether the 2005 or 2013 WHO severe pneumonia case definition was used. We did not explore schedules or previous use of PCV7, which may have had some influences on results. An additional limitation is the confounders associated with observational studies, such as changes to health care access or delivery, case definition, baseline pneumonia trends, pneumococcal nasopharyngeal carriage rates, and serotype distribution across settings.

## CONCLUSIONS

We identified important gaps in the existing literature, including the lack of PCV impact studies undertaken in setting with the highest under-five mortality and the lack of studies from the SEA region and LMICs in WPR. There is a need for large-scale studies in high mortality settings to demonstrate the evidence of vaccine impact on mortality.

There is clear evidence of declines in all-cause, severe, radiologically confirmed, bacteraemic pneumococcal pneumonia hospitalisations, as well as mortality across children of all age groups following PCV10 and PCV13 national introduction, although the magnitude was highly variable. These data support the ongoing use and introduction of PCV10 and PCV13 in countries that have not yet introduced PCV.

## Additional material


Online Supplementary Document
