# Supplementary material for: Systematic review on the impact of the pneumococcal conjugate vaccine ten valent (PCV10) or thirteen valent (PCV13) on all-cause, radiologically confirmed and severe pneumonia hospitalisation rates and pneumonia mortality in children 0-9 years old
Source: J Glob Health. 2023 Feb 3;13:05002. doi: 10.7189/jogh.13.05002 (PMC9896304; doi:10.7189/jogh.13.05002)
Supplement: Online Supplementary Document [file jogh-13-05002-s001.pdf]

ONLINE SUPPLEMENTARY DOCUMENT

Title: SYSTEMATIC REVIEW ON THE IMPACT OF THE PNUEMOCOCCAL CONJUGATE VACCINE TEN VALENT (PCV10) OR THIRTEEN VALENT (PCV13) ON ALL-CAUSE, RADIOLOGICALLY CONFIRMED AND SEVERE PNEUMONIA HOSPITALISATION RATES AND PNEUMONIA MORTALITY IN CHILDREN 0-9 YEARS OLD

Authors: Rita Reyburn[1], Anthea Tsatsaronis[2], Claire von Mollendorf[1, 3], Kim Mulholland[1, 3, 4] and Fiona M Russell[1, 3], for the ARI Review group.

Keywords: Pneumonia, Hospitalization, 10-valent pneumococcal conjugate vaccine, 13-valent pneumococcal conjugate vaccine, Pneumococcal conjugate vaccines, Children, Newborn, Infant, (Child, preschool), Mortality

Table S1. Search Strategy:

Medline (Ovid)

#	**Searches**
1	exp *Pneumonia/
2	((lower-respiratory adj3 infection*) or pneumonia or pneumonias or lung-inflammation* or lobitis or nonspecific-inflammatory-lung-disease* or peripneumonia or pleuropneumonia or pleuropneumonitis or pneumonic-lung* or pneumonic-pleurisy or pneumonic-pleuritis or pneumonitides or pneumonitis or pulmonal-inflammation* or pulmonary-inflammation* or pulmonic-inflammation*).tw,kf.
3	*pneumococcal infections/
4	*Streptococcus pneumoniae/
5	1 or 2 or 3 or 4
6	exp *Pneumococcal Vaccines/
7	exp *Immunization/
8	(pnu-im?une or pnuim?une or pcv10 or pcv-10 or pcv13 or pcv-13 or prevenar13 or prevenar-13 or prevnar13 or prevnar-13).tw,kf.
9	((10-valent or ten-valent or 13-valent or thirteen-valent) and (pneumococcal adj5 vaccine*)).tw,kf.
10	Immunization Programs/
11	6 or 7 or 8 or 9 or 10
12	*evaluation studies as topic/ or *program evaluation/
13	(impact or effectiveness or after or post or introduced or introduction).tw,kf
14	12 or 13
15	*hospitalization/ or *patient admission/ or *child, hospitalized/ or *inpatients/ or exp *mortality/ or *death/ or exp *infant death/ or *treatment outcome/
16	(inpatient* or admission* or mortalit* or death* or died or surviv* or fatal* or hospitali#ation or hospitali#ed).tw,kf.
17	exp *Outcome Assessment, Health Care/
18	15 or 16 or 17
19	(newborn* or new-born* or baby or babies or neonat* or neo-nat* or infan* or toddler* or pre-schooler* or preschooler* or kinder or kinders or kindergarten* or kinder-aged or boy or boys or girl or girls or child or children or childhood or youngster* or kid or kids or pediatric* or paediatric* or school-age* or schoolage* or schoolchild* or schoolgirl* or schoolboy*).tw,kf.
20	5 and 11 and 14 and 18 and 19

21	randomized controlled trial.pt.
22	exp randomized controlled trial/
23	exp case-control studies/
24	21 or 22 or 23
25	20 not 24
26	(exp animals/ or (rat or rats or mouse or mice or swine or porcine or murine or sheep or lamb or lambs or pig or pigs or piglet or piglets or rabbit or rabbits or cat or cats or dog or dogs or cattle or bovine or monkey or monkeys or trout or marmoset or marmosets).ti.) not human*.sh.
27	25 not 26
28	limit 27 to (case reports or comment or editorial or guideline or letter or practice guideline
29	27 not 28
30	limit 29 to yr="2003 -Current"

Cochrane Library

#	Searches
1	MeSH descriptor: [Pneumonia] explode all trees
2	((lower-respiratory-tract NEAR/3 infection*) OR pneumonia OR pneumonias OR lung-inflammation* OR lobitis OR nonspecific-inflammatory-lung-disease* OR peripneumonia OR pleuropneumonia OR pleuropneumonitis OR pneumonic-lung* OR pneumonic-pleurisy OR pneumonic-pleuritis OR pneumonitides OR pneumonitis OR pulmonal-inflammation* OR pulmonary-inflammation* OR pulmonic-inflammation* OR pneumococcal-infection* OR Streptococcus-pneumoniae) (Word variations have been searched)
3	MeSH descriptor: [Pneumococcal Infections] this term only
4	MeSH descriptor: [Streptococcus pneumoniae] this term only
5	#1 OR #2 OR #3 OR #4
6	MeSH descriptor: [Pneumococcal Vaccines] explode all trees
7	MeSH descriptor: [Immunization] explode all trees
8	(Immunization OR immunisation OR vaccination):ti,ab,kw
9	pnu-imune OR pnu-immune OR pnuimune OR pnuimmune OR pcv10 OR pcv-10 OR pcv13 OR pcv-13 OR prevenar13 OR prevenar-13 OR prevnar13 OR prevnar-13):ti,ab,kw
10	((ten-valent OR thirteen-valent) AND (pneumococcal NEAR/5 vaccine*)):ti,ab,kw
11	MeSH descriptor: [Immunization Programs] this term only
12	#6 or #7 or #8 or #9 or #10 or #11
13	MeSH descriptor: [Evaluation Studies as Topic] this term only
14	MeSH descriptor: [Program Evaluation] this term only
15	(evaluation OR impact OR effectiveness OR after OR post OR introduced OR introduction):ti,ab,kw
16	#13 or #14 or #15
17	MeSH descriptor: [Patient Admission] this term only
18	MeSH descriptor: [Hospitalization] this term only
19	MeSH descriptor: [Child, Hospitalized] this term only
20	MeSH descriptor: [Inpatients] this term only
21	MeSH descriptor: [Mortality] explode all trees
22	MeSH descriptor: [Death] this term only

23	MeSH descriptor: [Infant Death] explode all trees
24	MeSH descriptor: [Treatment Outcome] this term only
25	(inpatient* OR admission* OR mortalit* OR death* OR died OR surviv* OR fatal* OR hospitalisation OR hospitalization OR hospitalised OR hospitalized OR outcome*):ti,ab,kw
26	MeSH descriptor: [Outcome Assessment, Health Care] explode all trees
27	#17 or #18 or #19 or #20 or #21 or #22 or #23 or #24 or #25 or #26
28	(newborn* or new-born* or baby or babies or neonat* or neo-nat* or infan* or toddler* or pre-schooler* or preschooler* or kinder or kinders or kindergarten* or kinder-aged or boy or boys or girl or girls or child or children or childhood or youngster* or kid or kids or pediatric* or paediatric* or school-age* or schoolage* or schoolchild* or schoolgirl* or schoolboy*)
29	#5 and #12 and #16 and #27 and #28

Inclusion and exclusion criteria

Inclusion

- Post-licensure observational studies assessing PCV10/13 impact: Pre-PCV vs. PCV10/PCV13
- Study population aged 0-9 years old
- Study conducted > 3 years post-PCV introduction into the NIP
- 50% of the catchment population received PCV in the post-PCV period
- Hospitalisation due to pneumonia:
  > All cause pneumonia
  > Acute lower respiratory tract infection > > Severe pneumonia
  > Radiologically confirmed pneumonia
  > Clinical pneumonia
  > Laboratory confirmed invasive pneumococcal pneumonia (IPD pneumonia)
- Studies reporting on incidence rates, incidence rate ratios or percent decline
- Death due to pneumonia: definitions as above
- Death due to any cause

Exclusion

- Pre-licensure only
- RCTs, case-control studies, case reports and case series
- Comparison of PCV7 vs. PCV10/13 only
- Study does not include a comparison to the pre-PCV period / post-PCV 10/13 only

Appendix 3: Effective Public Health Practice Project (EPHPP) Quality Assessment Tool for Quantitative Studies; Assessment Results

Author	Selection bias (strong= representative & >80% participation; weak=not representative OR <60% participation OR neither are described)	Study design (strong=randomised trials; moderate=cohort/case control/time series; weak=all other)	Confounders	Data collection methods	OVERALL
**Alvarado 2018**	moderate	moderate	Not described	moderate	moderate
**Anderson 2017**	strong	moderate	Not described	moderate	moderate
**Andrade 2017**	moderate	moderate	moderate	moderate	moderate
**Andriatahirintsoa 2019**	neither described	moderate	Not described	moderate but poor description	moderate
**Becker-Dreps 2017**	strong	moderate	Strong	moderate	strong/moderate
**Ben-Shimmol 2018**	neither described	moderate	Not described	moderate	moderate
**Ben-Shimol 2017**	moderate	moderate	moderate	moderate	moderate
**Berger 2019**	neither described	moderate	Strong	moderate	moderate
**Camargos 2020**	neither described	moderate	Not described	moderate	moderate
**Chacon-Cruz 2019**	neither described	moderate	moderate	moderate	moderate
**Congdon 2020**	moderate	moderate	Strong	moderate	moderate
**DeOliveira 2020**	neither described	moderate	Strong	moderate	moderate
**Dondo 2019**	neither described	moderate	moderate	moderate	moderate
**Faye 2019**	moderate	moderate	Not described	moderate	moderate
**Haji 2018**	neither described	moderate	Not described	moderate	moderate
**Hammitt 2019**	strong	moderate	Strong	Strong	Stong
**Izu 2017**	neither described	moderate	Strong	moderate	moderate
**Jimenez-Trujillo 2017**	moderate	moderate	Strong	moderate	moderate
**Kabore 2020**	moderate	moderate	Strong	moderate	moderate

**Kupek 2016**	neither described	moderate	Not described	moderate	moderate
**Laaksonen 2016**	neither described	moderate	moderate	moderate	moderate
**Luca 2018**	neither described	moderate	moderate	moderate	moderate
**Mackenzie 2017**	strong	moderate	Strong	Strong	Stong
**Meder 2020**	neither described	moderate	Not described	moderate	moderate
**Mpabalwani 2019**	strong	moderate	Strong	moderate	strong/moderate
**Naucler 2019**	strong	moderate	Strong	moderate	strong/moderate
**Palmu 2017**	strong	moderate	Strong	Strong	Strong
**Petousis-Harris 2019**	neither described	moderate	moderate	moderate	moderate
**Ruvinsky 2018**	strong	moderate	Strong	moderate	strong/moderate
**Saxena 2015**	strong	moderate	Strong	moderate	strong/moderate
**Sgambatti 2016**	strong	moderate	Strong	moderate	strong/moderate
**Sigurdsson 2020**	neither described	moderate	moderate	moderate	moderate
**Silaba 2019**	strong	moderate	Strong	Strong	Strong
**Silva 2016**	strong	moderate	Strong	moderate	strong/moderate
**Thorrington 2018**	moderate	moderate	moderate	moderate	moderate
**Triadou 2020**	neither described	moderate	Not described	moderate	moderate
**van Deursen 2017**	Weak	moderate	Not described	moderate	moderate
**Vestjens 2019**	moderate	moderate	Not described	moderate	moderate
**Wiese 2016 (empyema)**	neither described	moderate	Strong	moderate	moderate
**Wiese 2016 (pneumonia)**	neither described	moderate	Strong	moderate	moderate
**Zampoli 2015**	strong	moderate	moderate	moderate	moderate
**Schuck-Paim 2019**	strong	moderate	Strong	moderate	strong/moderate
**Takeuchi 2020**	neither described	moderate	moderate	moderate	moderate

Table S2. Characteristics of the 43 studies included in this review													
**Author**	**Country**	**Study Design**	**Study Population**	**Setting**	**No. of cases**	**Age**	**PCV Type, Schedule, Catch up**	**Date of PCV introduction**	**Time Period of Analysis**	**Years Post-PCV10/13**	**Vaccine Coverage (%)**	**Pneumonia Definition**	**Data Source**
**Low Income Status**													
Kabore 2020 **[39]**	Burkina Faso	Retrospective hospital-based record review cohort study: Interrupted time-series analysis	Children <5 years living in catchment area of 4 health districts: Séguénéga, Nouna, Orodara and Ndorola in Burkina Faso	4 district hospitals	5,771	<5 years	PCV13, 3+0, no	PCV13 Oct 2013	Pre-PCV: Jan 1 2009 - Oct 31st 2013<br>PCV introduction period: Nov 2013 - 31st Dec 2014<br>Post-PCV: Jan 1 2015 - Dec 31st 2018	5	2015, the administrative coverage for three PCV doses was 108.2% in Nouna, 97.7% in Orodara, and 108.3% in Séguénéga health districts	All-cause pneumonia: patient with a clinical diagnosis of acute lower respiratory infection (ALRI), severe ALRI, pneumonia, severe pneumonia, bronchopneumonia, severe bron-chopneumonia, bronchiolitis, or pleural effusion (Gatera et al., 2016) at hospital admission or discharge.	Hospital admission records - patient charts and hospitalization logbooks

												Severe pneumonia (World Health Organization, 2005)	
Hammitt 2019 [56]	Kenya	Prospective hospital based, cohort study; Pre/Post comparison	Residents of Kilifi Health and Demographic Surveillance System (KHDSS)	Kilifi County Hospital admissions	3,211403 *all ages	All ages, including children <5 and children between 5-17 years	PCV10, 3+0, yes, one dose for children <5 years	PCV10 Jan 2011	Pre-PCV: Jan 1 1999-Dec 31st 2010 Post-PCV: Jan 1 2012-Dec 31st 2016	5	At least 2 doses PCV10 ages 2-11months 2011: 80% 2016: 84% At least 1 dose in children aged 12-59months" 2011: 66% 2016: 87%	Pneumococcal pneumonia: case of IPD in a child with cough or difficulty breathing, and at least one of the following: lower chest wall indrawing, central cyanosis, inability to drink, convulsions, lethargy, prostration or head nodding	Integrated clinical, laboratory and demographic surveillance system
Andriatahirintsoa 2019 **[14]**	Madagascar	Retrospective hospital-based cohort study; pre-post	Children <5 years admitted to CHUMET	Centre Hospitalier Universitaire Mere Enfant Tsaralalana (CHUMET),	4,974	<5 years	PCV10, 3+0, not stated	PCV10 Oct 2012	Pre-PCV10: 1 Jan 2010 - 31 Dec 2011	5	Madagascar national coverage 2013: 76% 2014: 72% 2015: 69%	Clinical pneumonia: diagnosis of pneumonia by examining clinician	Hospitalization admission logbooks from 2010 to 2017

		comparison		pediatric hospital					Year of PCV10 introduction: 2012<br><br>Post-PCV10: 1 Jan 2013-31 Dec 2017		2016: 74%<br>2017: 74%		
Izu 2017 [24]	South Africa	Retrospective hospital-based cohort study; time series	HIV-infected or un-infected children <5 years in Soweto, South Africa	Chris Hani Baragwanath Academic Hospital, Soweto, South Africa	26,778	<5 years	PCV13, 2+1, Yes children <30 months	PCV7 April 2009<br><br>PCV13 replacement in May 2011	Pre-PCV: Jan 2006-2008<br><br>PCV7: 2010-2011<br><br>PCV13: 2012-Dec 2014	3.5	National coverage with 3rd dose in children 9 months of age<br><br>2009: 10,4%<br><br>2010: 64.3%	All-cause pneumonia (ICD-10)<br>Codes:<br>B05.2, B20.6, B25, B59, J10, J12,<br>J12.1, J12.2, J12.8, J12.9, J13, J14, J15,<br>J15.1, J15.2, J15.3, J15.4, J15.5, J15.8,<br>J15.9, J16.8, J17, J18 and/or J18.1.	Electronic database covering admissions to general pediatric medical ward at study hospital

											2011: 89.8%<br><br>2012: 99.0%	P36.0, P36.1,P36.2, P36.4, P36.8 and/or P36.9	
Mackenzie 2017 [41]	The Gambia	Prospective population-based surveillance cohort study;	Children aged 2–59 months between May 12, 2008- Dec 31, 2015<br><br>All residents of The Gambia aged 2 - 59 months	All outpatients and inpatients at all health facilities in the BHDSS<br>Basse Health and Demographic Surveillance System (BHDSS)[1]	Clinical pneumonia: 18 833<br><br>Radiologically confirmed pneumonia: 2156	2-59 months	PCV13, 3+1, no	PCV7 Aug 2009<br><br>PCV13 May 2011	Pre-PCV13: May 12 2008 - May 11 2010<br><br>PCV13: Jan 1, 2014 - Dec 31, 2015	3.5	95% children born in the last 6 months of 2014 received 2 or more doses of PCV13 before age 12 months,<br><br>Coverage of at least two doses of PCV13 in the 2–23 month age	Clinical pneumonia: cough or difficulty breathing for less than 14 days accompanied by one or more of raised respiratory rate for age, lower chest wall indrawing, nasal flaring, grunting, O2 saturation less than 92%, altered consciousness, inability to sit or feed, convulsions, dull chest percussion note, coarse crackles, or bronchial breathing	Clinicians recorded clinical findings and applied standardised criteria in accordance with a standardised protocol

[1] Study population included both outpatients and inpatients, however vaccine impact estimates were unchanged in stratified analyses excluding outpatients

											group plateaued at about 70% in early 2014	Radiologically confirmed pneumonia (WHO-defined)<br><br>Pneumococcal pneumonia – Radiological: WHO defined radiologically confirmed pneumonia plus isolation of *S pneumoniae* from a sterile site<br><br>Pneumococcal pneumonia – Clinical: defined as for clinical pneumonia with the addition of isolation of *S pneumoniae* from a sterile site;	

												Clinical pneumonia – hypoxic: pneumonia, defined as clinical pneumonia with peripheral O2 saturation less than	
**Lower-Middle Income Status**													
Silaba 2019 [42]	Kenya	Prospective hospital and population surveillance	Residents aged ≥2 months to <12 years of the Kilifi Health and Demographic Surveillance System (KHDSS)	Kilifi County Hospital (Kilifi, Kenya) is centrally located within KHDSS and is the only paediatric inpatient facility in the study area.	Clinical pneumonia: 8488	2months – <12 years	PCV10, 3+0, Yes to 12–59 months	PCV10 Jan 2011	Clinical pneumonia<br>Pre PCV: May 2002-Dec 2010<br>Transition period: Jan -March 2011<br>Post PCV: April 2011 -	4	2–11 months (≥2 doses)<br>2011: 79·9<br>2012: 76·4<br>2013: 81·4<br>2014: 87·7<br>2015: 84·2<br>12–23 months (≥1 dose)	Clinical pneumonia – severe or very severe (WHO)<br>Radiologically confirmed pneumonia (WHO-defined)	Kilifi Health and Demographic Surveillance System (KHDSS)

			2002, there were 37 556 residents in KHDSS<br>aged 2–59 months and 44 672 residents aged<br>60–143 months. By March, 2015, these figures were 45 601<br>and 62 502, respectively.						March 2015<br><br>Radiologically confirmed pneumonia<br><br>Pre-PCV: April 2006 – Dec 2010<br><br>Transition period: Jan -March 2011<br><br>Post-PCV: April 2011 – March 2014		2011: · 76·0<br>2012: 84·4<br>2013: 85·6<br>2014: 89·3<br>2015: 91·6<br><br>24–59 months (≥1 dose)<br>2011: 62·7<br>2012: 66·9<br>2013: 74·6<br>2014: 82·8<br>2015: 86·5<br><br>60–143 months (≥1 dose)<br>·2011: 7·2<br>2012:15·7		

											2013: 24·1<br>2014: 32·4<br>205: 42·8		
Becker-Dreps 2017<br>[15]	Nicaragua	Retrospective population based cohort study	All residents from all age groups in<br>Leon, Nicaragua<br><br>2015 total population = 410860	All 107 public health facilities in Leon Department, Nicaragua	Average age /week 0-1 year olds: 56-90	All ages, including children <5 years	PCV13, 3+0, yes	PCV13 Dec 2010	Pre-PCV: 2008-2010<br><br>Transition year: 2010<br><br>Post-PCV/PCV13: 2011-2015	5	2011: 63% of infants<br><br>87% of 1 year old had catch up dose<br><br>2012: 97% infants<br><br>Since 2012: range of 89%-100% by municipality	All-Ccuse pneumonia: infectious syndrome including constitutional and respiratory symptoms, present with physical exam findings of consolidation, with or without conformation of infiltrate on chest radiograph<br><br>Pneumonia -related death: patient dies of pneumonia in any health facility	Office of Vital Statistics in the Department of Leon<br><br>Hospital epidemiology database

Faye 2019 [23]	Senegal	Retrospective hospital-based cohort study; Interrupted time series analysis	Children aged <5 years admitted for pneumonia between Oct 2010-Oct 2016	CHNEAR - Large national and subregional tertiary peadiatric hospital in Dakar	1,836	<5 years	PCV13, 3+0, no	PCV13 Oct 2013	Pre PCV: Oct 2010-Sep 2013<br><br>Transition period: Oct 2013-Sep 2014<br><br>Post-PCV: Oct 2014 - Oct 2016	3	National coverage for 3 doses was 81% in 2014 and 93% in 2016<br><br>Coverage rates for Dakar region range: 75% (2015) - 79% (2016)	Clinical pneumonia: based on keywords identified in ward logbooks, bronchiolitis was excluded	Logbooks of 4 pediatric wards
Mpabalwani 2019 [28]	Zambia	Retrospective population based cohort study; Time Series Analyses	Total population of children <5 years in Zambia, approximately 2.2 million	Urban health centres<br>that reported to Ministry of Health (MOH)<br>AND	165,717	<5 years	PCV10, 3+0, no	PCV10 July 2013	Pre-PCV:<br>Jan 2010–June 2013<br><br>Transition period: July 2013-June 2014	3.5	Coverage for 3 doses in Zambia:<br>2014 - 77%<br>2016 – 90%	All-cause pneumonia: all “respiratory infection pneumonia”<br>And all-cause pneumonia code J18.9 (ICD-10)	Hospital administrative data

				The University Teaching Hospital (UTH)					Post PCV: July 2014–Dec 2016				
Dondo 2019 [22]	Zimbabwe	Retrospective hospital-based cohort study; time series	Greater Harare area : Catchment population of HCH 2.1 million in total (all ages)	Harare Central Hospital (HCH)	Average/month: 1,330	<5 years	PCV13, 3+0, no	PCV13 in July 2012	Pre-PCV: 1st Jan 2010-June 2012 Vaccine uptake period: July 2012-June 2013 Post-PCV13: July 2013-31st Dec 2016	3.5	National coverage rates 2013-2016 Range: 87%-92% Harare coverage rates range: 84% - 104%	All cause pneumonia (ICD-9 and ICD10)	Hospital discharge Data
**Upper and Lower-Middle Income Status**													

de Oliveira 2020 [21]	10 Latin American and Caribbean Countries: Argentina, Brazil, Colombia, Ecuador, Honduras, Mexico, Nicaragua *Dominican Republic,	Retrospective hospital-based cohort study; time series	Children <5 years in 10 Latin America and Caribbean Countries	Nationwide	Not stated	<5 years	Argentina: PCV13, , 2+1, not stated Brazil: PCV10, 2+1, Yes, 2 +1 doses for 7-11 months of age, and 1 dose for 12-23 months of age Colombia: PCV10, 2+1, not stated	Argentina: PCV13 Jan 2012 Brazil: PCV10 2010 Colombia: PCV10 Sep 2011 Ecuador: PCV10 in Aug 2010 Honduras: PCV13	2000-2016 POST-PCV periods reported only: Argentina : Jan 2012 - Dec 2015 Brazil: Mar-2010 to Dec-2015 Colombia: Nov-2011 to Dec-2015 Ecuador: Aug-2010 to Dec-2016	Argentina :4 Brazil: 5 Colombia :4 Ecuador:6 .5 Honduras: 5.5 Mexico: 4.5	Not stated	All-cause pneumonia: ICD-10 code: J12-J18 as primary cause of death)	national mortality registries in the LAC region

	Guyana and Peru excluded as do not individually meet inclusion criteria						Ecuador: PCV10, 3+0, not stated<br><br>Honduras: PCV13, 3+0, not stated<br><br>Mexico: PCV7 and PCV13, 2+1, not stated<br><br>Nicaragua: PCV13, 3+0, yes	April 2011<br><br>Mexico: PCV7 in 2008<br>PCV13 May 2012<br><br>Nicaragua: PCV13 Dec 2010	Honduras: Jan-2011 to Dec-2016<br><br>Mexico: Feb-2008 to Dec-2016<br><br>Nicaragua: Jan-2012 to Dec-2015	Nicaragua: 5			

**Upper-Middle Income Status**													
Ruvinsky 2018 [51]	Argentina	Observational, prospective, population-based surveillance study	Children <5 years of age in the Department of Concordia, Argentina from April 2014 – March 2016<br><br>Concordia:	The Delicia Concepción Masvernat General Hospital - with a catchment population of all Concordia residents. More than 95% of hospitalized patients are served	1,098	<5 years	PCV13, 2+1, not stated	PCV13 Jan 2012	Pre-PCV13: Nov 2002-Oct 2005 (Data from a previous study (14) in Concordia (2002 – 2005) was used as a baseline to evaluate PBP incidence)	4	In this study population<br><br>PCV13 coverage with dose 1 was 97.3% (95%CI: 96.0 – 99.4), decreasing to 84.8% (95%CI: 79.1 –	Radiologically confirmed pneumonia (WHO-defined)	Hospital based surveys, interviews and medical history reports

			<5 years = 15, 493<br><br>< 2 years: 6, 500	by this hospital. And 2x abulatory care hospitals and 1 health care center					Post-PCV13:<br>1 April 2014 – 30 March 2016		90.5) by the booster dose.		
Andrade 2017<br>[13]	Brazil	Retrospective hospital-based cohort study; interrupted time series	Children < 10 years old in Brazil	Nation wide	2, 053419	< 10 years	PCV10, 3+1, Yes, 2 +1 doses for 7-11 months of age, and 1 dose for 12-23 months of age	PCV10 March -Sep 2010	Pre-PCV10: Jan 2005 – Dec 2009<br><br>Post-PCV10: 2011-2015	5	Vaccine coverage for three primary doses:<br>2011: 81.7%<br>2012: 88.4%<br>2013: 93.6%<br>2014: 92.9%<br>2015: 94.2%	All-cause pneumonia (ICD10)	Population based data from National hospitalization Information System (SIH)

Camargos 2020 [18]	Brazil	Ecological study	Brazilian children <5 years	Nationwide	387, 201	<5 years	PCV10, 3+1, Yes, 2 +1 doses for 7-11 months of age, and 1 dose for 12-23 months of age	PCV7 2002<br><br>PCV10 March -Sep 2010	Jan 1990-Dec 2017	7	Three doses of PCV10 coverage:<br>2010: 24%<br>2011: 82%<br>2012: 88%<br>2013: 94%<br>2014: 93%<br>2015: 94%<br>2016: 95%<br>2017: 91%<br><br>Average coverage of 91% from 2011-2017	Death due to lower respiratory infections (LRI) as indicate by the clinician on the death certificate[2]	Database for national vital register which includes cause of death as decided by the attending clinician
da Silva 2016 [20]	Brazil	Retrospec tive populatio	All Children < 1 year living in 26	26 municipaliti es of Brazil	5,04 4	< 1 year	PCV10, 3+1, Yes, 2	PCV10 March 2010	Pre-PCV: 2007-2009	3	2010: 52.72 %	Community-acquired pneumonia (CAP)	Tabwin Database, regional

[2] Full case definition described in their previous study: GBD 2017 causes of death collaborators. Global, regional, and national age-sex specific mortality for 282 causes of death in 195 countries and territories,1980–2017: a systematic analysis for the Global Burden of Disease Study 2017. Lancet 2018; 392: 736–88.

		n-based cohort study; Pre- post comparison	municipalities under the jurisdiction ofSRS/Alfenas----State Department of Health of Minas Gerais,Brazil between 2007-2013<br><br>Mean no. children < 1 in the 26 municipalities pre vaccine period: 5740, post vaccine period: 5686				+1 doses for 7-11 months of age, and 1 dose for 12-23 months of age		PCV10 introduction: 2010<br><br>Post-PCV10: 2011-2013		2011: 102.99 %<br><br>2012: 102.88 %<br><br>2013: 103.10 %		health authority hosptial admission databse
Kupek 2016 [26]	Brazil	Retrospective populatio	Children <5 years in the state of	Hospitals<br>293 municipaliti	75,891	<5 years	PCV10, 3+1, Yes, 2	PCV10 March 2010	Pre-PCV: 2006-2009	4	2010 only 3.4% of	Pneumonia: (ICD-10, codes J12-18)	Hospital Information

		n-based cohort study; time series	Santa Catarina, Brazil	es of Santa Catarina, Brazil			+1 doses for 7-11 months of age, and 1 dose for 12-23 months of age		Introduction period: 2010<br><br>Post-PCV: 2010-2014		municipalities achieved coverage of 95% or more;<br><br>From 2011-2014, 60% of municipalities acheived coverage > 95%		System of the Brazilian National Health System - SIH/ SUS)
Schuck-Paim 2019 [58]	Brazil	Retrospective population -based cohort study: Time series	Children aged 3–59 months in Brazil	Nationwide	1459	3-59 months	PCV10, 3+1, Yes, 2 +1 doses for 7-11 months of age, and 1 dose for 12-23 months of age	PCV10 2010	Pre-PCV: April 1, 2004 - March 31, 2009<br><br>Post-PCV: April 1, 2010 - March 31, 2014	4	Mid-2012 = coverage reached high levels (80–85% of the target population)	Pneumonia mortality: All-cause pneumonia: ICD-10 codes J12–18	Publicly available mortality data

Sgambatti 2016 [33]	Brazil	Prospective population-based surveillance studies	children aged 2- 35 months Living in Goiânia municipality, capital of Goiás state, located at the Central-Western Region of Brazil 52, 562 in 2012	All paediatric hospitals in Goiânia municipality.	8,191	2-35 months	PCV10, 3+1, Yes, 2 +1 doses for 7-11 months of age, and 1 dose for 12-23 months of age	PCV10 June 2010	Pre-PCV: May 2007 - Apr 2009 Post-PCV: Nov 2011- Oct 2013	3	PCV10 complete 3-doses 2–11: 2011: 93.3%, 2012: 91.3% 2013: 92.0%, Overall <12 months of age = 58%. In Goiânia municipality	Clinical pneumonia: defined as any case of suspected CAP, diagnosed in the first 72 h of hospital admission, and recorded on the medi-cal charts, irrespective of the causal agent Radiologically confirmed pneumonia (WHO-defined)	medical charts review and interviews of parents or legal guardians
Congdon 2020 [19]	Botswana	Retrospective hospital	Children 1-59 months of age	Three Hospitals in Botswana	Pneumonia	1-59 months	PCV13, 3+0, no	PCV13 July 2010	Pre-PCV: Jan 2009 - Oct 2010	6.5	Vaccine series coverage	Pneumonia defined as: 'pneumonia',' lower respiratory	Written admission

		based cohort study; Interrupted time-series analysis	living in Botswana between Jan 2009-Dec 2017		hospitalisations: 6943<br><br>Pneumonia-related deaths: 201				PCV Introduction: Oct 2010 - Jan 2013<br><br>Post-PCV: Jan 2013 - Dec 2017		rates estimated to be 81-95%	infection', 'respiratory tract infection', 'lower respiratory tract infection', 'bronchitis', 'bronchiolitis', 'PCP', 'aspiration pneumonia', 'bronchopneumonia' or 'pulmonary tb'	and ward registers
Alvarado 2018 [11]	Chile	Retrospective hospital-based cohort study; pre-post comparison	Children <5 years of age in southern area of Santiago - population of 17 communes	Four hospitals accepting children referrals from 17 Communes in the southern area of Santiago	6,461	<5 years	PCV10 , 2011: 3+1 and 2013 onwards: 2+1, no	PCV10 in Jan 2011	Pre-PCV: 2009-2010<br><br>Post-PCV: 2011-2015	4	Not stated	Community acquired pneumonia (CAP) (ICD-10 codes: J13 - J18)	Clinical record and Hospital discharge records

			Total population of children <5 years 2009-2015 =1,359,652										
Chacon-Cruz 2019 [47]	Mexico	Prospective hospital-based cohort study	All children < 16y with cases of pleural empyema at General Hospital of Tijuana, Mexico	General Hostapal of Tijuana, Mexico	64	< 17years	PCV13, 2+1, not stated	PCV13 May 2012	Pre-PCV: Oct 2005-April 2012<br><br>Post-PCV: May 2012 - Jan 2018	6	Not stated	Pleural empyema: cases with clinical/radiologically confirmed diagnosis of community acquired pneumonia complicated by pleural effusion<br><br>Pneumococcal pleural empyema: pleural empyema with detection of *S.pneumoniae* serotypes by either Quellung reaction or PCR in pleural fluid	Data collection form from active/prospective surveillance
Zampoli 2015	South Africa	Retrospective hospital-	children <12 years	Paediatric referral hospital in	164	<12 years	PCV13, 2+1, yes	PCV7 April 2009	Dec 2006 - Dec 2014	3	Not stated	Empyema: pleural effusion that after pleural tap was	Hospital admissions database

[49]		based cohort study; Pre- post comparison	admitted with empyema at a tertiary paediatric hospital in Cape Town, South Africa, from December 2006 to December 2011 (cohort A) and January 2012 to December 2014	Cape Town, South Africa				PCV13 July 2011	Pre-PCV13 (Cohort A): Dec 2006 - Dec 2011<br>Post-PCV13 (Cohort B): Jan 2012- Dec 2014			purulent or turbid on inspection or showed neutrophil predominance on cell count.	and the pulmonology service records
**High Income Status**													
Meder 2020<br>[57]	Australia	Retrospective population-based cohort	All children (indigenous and non-indigenous) = or < 4	Nationwide	Not stated	All ages, including children	PCV13, 3+0<br>no	PCV7 Jan 2005	Pre-PCV: 1 July 2002–30 June 2004	5 years	From 2005, 3-dose<br>PCV coverage at 12 months	Pneumococcal community-acquired pneumonia without IPD (non invasive) (PnCAP):	Australian Institute of<br>Health and Welfare's National

		study; Pre- post comparis on	years old in Australia during the period of : **1st July 2002-30 June 2016**			n < 4 years		PCV13 July 2011	Early PCV7:2005 –2007<br><br>Pre-PCV13: 2008 to mid- 011<br><br>Post-PCV13: mid-2011-2016		of age was around 90% in both Indigenous and non-Indigenous children	(ICD-10-AM) discharge codes J13 and/or J18.1 , excluding cases with G00.1 and A40.3 codes	Hospital Morbidity Database
Anderson 2017 [12]	Canad a	Retrospec tive populatio n based cohort study	Children aged 6-59 months old in Quebec province:<br><br>2000: 359,003<br><br>2015:401,5 74	All acute care facilities in province of Quebec	63,1 08	6-59 month s	PCV13, 2+1 and 3+1, not stated	PCV7 June 2005 PCV10 replac ement of PCV7 in Oct 2009	Pre-PCV: 1st April 2000 – 2004<br><br>PCV7: 2004-2009<br><br>PCV10: 2009-2011	45po st PCV1 0<br><br>4 PCV1 3	> 94% since 2004	Pneumonia: (ICD-9; 480.x, 481, 482.x, 483, 484.x, 485, 486, 487.0 & ICD-10; J10.0, J11.0, J12.x, J13, J14, J15.x, J16.x, J17.x, J18.x)<br><br>Empyema (ICD-9; 511.x & ICD-10; J90)	Med-Echo, hospital discharge database of the Quebec Health insurance board

								PCV13 replacement of PCV10 in Nov 2010	PCV13: 2011- 31 Dec 2014				
Haji 2018 [40]	Canada	Retrospective hospital-based cohort study	Children < 18 years living in the Champlain Local Health Integration network (CLHIN) over study period approx. 202,358-370,408	Children's Hospital of Eastern Ontario (CHEO	371	< 18	PCV13, 2+1 and 3+1, not stated	PCV7 in 2005<br>PCV10 replaced pCV7 in 2009<br>PCV13 replaced PCV10 in late 2010	Pre-PCV: 2002-2004<br>PCV7/Pre-PCV13: 2005-2011<br>Post-PCV13: 2012-2015	5	Not stated	Pediatric complicated pneumonia (PCOMP): included empyema, parapneumonic effusion, necrotising pneumonia and lung abscess (ICD-10-CA)	Medical records, focusing on discharge summaries

Luca 2018 [27]	Canada	Retrospective hospital-based cohort study	All Ontario residents eligible for Ontario Health Insurance Plan [OHIP] between April 1992 and March 2014	All acute care hospitalizations in Ontario	1,063,700 *all ages	All ages, including children < 2 years and children 5-17 years	PCV13, 2+1 and 3+1, not stated	PCV7 June 2005 PCV10 replacement of PCV7 in Oct 2009 PCV13 replacement of PCV10 in Nov 2010	Pre-PCV: April 1992-May 2001 PCV7 (private purchase): June 2001 – Dec 2004 PCV7 (public funding): Jan 2005 - Sep 2009 PCV10: Oct 2009 - Oct 2010 PCV13: Nov 2010-	4.5 years post PCV10 or PCV13 (PCV10 was introduced for 1.5 years and then replaced by PCV13)	Not stated	Pneumonia (ICD-9 & ICD-10-CA)	Discharge Database of the Canadian Institute for Health Information (CIHI)

									March 2014				
Saxena 2015 [32]	England	Retrospective population-bases cohort: Interrupted time series	children < 16 years in England	Nationwide	Pneumonia: 172,066 Empyema: 3828	< 16 years	PCV13, 1+1, no	PCV7 Sep 2006. PCV13 replacement in April 2010	Pre-PCV: 1st April 2001 - 31st Aug 2006 PCV7: 1st Sep 2006 - 31st March 2010 PCV13: 1st April 2010 - 31st March 2014	4	PCV13 uptake in UK: Uptake during 2010/11 was 94% for children at 12 months 91% for the booster at 13 months	All-cause pneumonia (ICD-10 codes J12-18) Empyema (ICD-10 codes J86.0, J86.9)	The Hospital Episodes Statistics (HES) database
Thorrington 2018 [35]	England	Retrospective population and laboratory based cohort; timer series	Individuals of all ages in England	Nationwide	Pneumococcal pneumonia: 30,459 Empyem	All ages, including children < 4 years and -	PCV13, 2+1, no	PCV7 Sep 2006 PCV13 replaced in	Pre-PCV: 1 April 2004 - 31 March 2006 Post-PCV: 1 April 2013 -	5	Not stated	Pneumococcal pneumonia: ICD10 code J13 Empyema: ICD-10 code J869	Hospital Episodes Statistics (HES) Data

		using composite controls			a: 23,434<br>Pneumonia with lung abscess: 2,616<br>Pneumonia unspecified organism: 30,459<br>*all ages	14 years		April 2010	31 March 2015			Abscess of lung with pneumonia: ICD-10 code J851<br>Pneumonia unspecific organism - ICD10 code: J18	
Laaksonen 2016 [50]	Finland	Retrospective hospital-based	population of approx. 90,000	Tampere University Hospital,	202	< 36 months	PCV10, 2+1, not stated	PV10 Sep 2010	Pre-PCV: 2008–2009	3	Not stated	Radiologically confirmed pneumonia: Children with fever and blood leucocytes	Hospital records

		cohort study;	children < 16 years	Tampere, Finland.					Post-PCV: 2012–2013.			of >17.5 9 10E9/L and with pneumonic infiltrate on radiograph, excluded the cases with an infection focus – except pneumonia and otitis media	
Palmu 2017 [30]	Finland	Retrospective population-based, cohort study	Two cohorts: "Target Cohort" Vaccine eligible children = born from June 1 2010 to September 2013- Follow up started from 3months and ended between	Nationwide	334 087 child - years of follow-up in the target cohort eligible for vaccination	3 - 71 months	PCV10, 2+1. no	PCV10 Sep 2010	Pre-PCV1 (Reference cohort 1 & 2) : 2003-2008<br><br>Post-PCV1 (target cohort): June 2010-Sep 2013<br><br>Indirect cohort = vaccine ineligible	3	Children born in 2012:<br>93% for the first dose and 92% for the full series of three doses	Hospital Ddagnosed pneumonia (HDP)ICD-10 codes: J10.0, J11.0, J12-J18, J85.1 or J86<br><br>Hospital-treated primary pneumonia (HTPP): diagnosis of pneumonia after in-patient hospitalization<br><br>Pneumococcal pneumonia (ICD-10) code J13	Hospital discharge register

			ages of 3-42months<br><br>"Target cohort for indirect effects" - Not eligible for vaccine = older children born between Jan 2008 - May 2010- followed up between 7 to 71 months of age<br><br>Both compared with two season and age						Pre-PCV2 (indirect refrence cohort (2004-2008)<br><br>Post-PCV2 (indirect target cohort): 2011-2013 (follow up period)<br><br>These are cohort follow up periods			Empyema: ICD10 code J86 and hospitalization at least overnight.<br><br>Bacterial pneumonia: positive blood culture or the isolation of microorganisms from washed sputum samples.	

			matched reference cohorts										
Sigurdsson 2020 [34]	Iceland	Retrospective hospital-based cohort study	2 cohorts children born between 2005-2015<br><br>51,264 children were followed<br><br>All Icelandic children born 2005–2015 were followed from birth	Primary paediatric hospital in Iceland	660	< 36 months	PCV10, 2+1, no	PCV10 in April 2011	2005-2010 birth-cohorts were defined as vaccine non-eligible cohorts (VNEC)<br><br>2011–2015 birth cohorts as vaccine eligible cohorts (VEC)	5.5	> 97% of children receiving the two primary doses <12 months in 2011	Pneumonia codes J09-J18 (ICD10)	The Children's Hospital Iceland - hospital inpatient registry

			until three years of age, death, emigration or the end of the study period.										
Ben-Shimol 2018 [55]	Israel	Prospective population based, cohort study; Pre/Post comparison	All children <5 years old in Israel = 850,000 in 2014	All 27 medical health centers in Israel *26 hospitals admitting children and one major outpatient health maintenance organisation	1,478	<5 years	PCV13, 2+1, no	PCV7 July 2009 PCV 13 replacement Nov 2010	Pre-PCV: July 2000-2008 PCV7: 2009-2011 PCV13: 2014- June 2016	5.5	June 2011: 80% of children 7-11month received > or = 2 doses of PCV7 and /or PCV13 Dec 2012: 90% of children 7-11month received > or = 2 doses of	Pneumococcal pneumonia: illness with positive blood culture for S. pneumoniae with clinical diagnosis of pneumonia (physician diagnosis)	Nationwide active surveillance monthly questionnaires

											PCV7 and /or PCV13<br><br>June 2014: 95% children 7-11months had = > 2 doses of PCV13<br><br>June 2011: 36% of children 24-35months received > or = 2 doses of PCV7 and /or PCV13<br><br>Dec 2012: 87% of children 24-35		

											month received > or = 2 doses of PCV7 and /or PCV13<br><br>June 2014: 91% children 24-35 months had = > 3 doses of PCV13		
Berger 2019 [17]	Israel	Retrospective hospital-based cohort stud; pre-post comparison	Children < 18 with no history of chronic underlying disease hospitalized for community acquired bacteremia at three	Three tertiary pediatric hospitals in Israel	125, 92	< 18 years	PCV13, 2+1, no	PCV13 in Nov 2010	Pre-PCV13: Jan 1st 2007 – Dec 2009<br><br>Post-PCV: Jan 2010 – Dec 31st 2015	5	Coverage in Israel in 2010: 81%<br>2011: 90%<br>2012: 89%<br>2013: 89%	Lower respiratory tract infection (LRTI): pneumonia and/or bronchiolitis. Clinical diagnosis including fever, respiratory complaints, shortness of breath, coughing and wheezing	Administrative records in the three tertiary hospitals

			children's hospitals in Tel Aviv and Jerusalem										
Triadou 2020 [53]	Israel	Prospective, population-based cohort study	Approxi. 75,000 children <60 months in Negav district of Southern Israel	Soroka University Medical Centre is the only hospital in the Negev district of southern Israel	12,271	Children <60months	PCV13, 2+1, no	PCV7 July 2009<br>PCV13 replacement Nov 2010	Pre-PCV: July 2002–June 2008<br>PCV7: July 2010–June 2011<br>PCV13: July 2013–June 2016.	5.5	95% in Negev<br>80% of children 7–11 months old had received 2 doses of PCV7, and by Dec 2012, 90% had received 2 doses of PCV13. By June 2014, 95% of children	Radiologically confirmed pneumonia (WHO-defined) with (PE-CAP) or without (NPE-CAP) pleural effusion	The Soroka University Medical Centre

											24–35 months old had received 2 PCV13 doses		
Takeuchi 2020[3] [52]	Japan	Hospital based surveillance cohort study[4]	Children aged 1 month to 15 years who lived in Chiba City and were admitted to hospital with CAP Population of children	Hospital-based, 15 hospitals in Chiba City,central Japan.	1,299	= or < 15 years	PCV13, 3+1, not stated	PCV7 available from 2011, in NIP April 2013 PCV13 replacement Nov 2013	Pre-PCV: 2008 PCV7: 2012 PCV: April 2016 to March 2019	5	Not stated for PCV13 PCV7: 95%	Radiologically confirmed pneumonia: At least one of the following abnormal clinical findings on chest radiograph: fever, cough, rapid breathing, difficulty in breathing, or crackles on auscultation of the lungs.[5]	Primary surveillance data Collected and recorded on standard case report sheet

[3] Data from Pre-PCV and PCV7 periods are from their previous studies

[4] Unclear if retrospective or prospective

[5] This definition is unclear, it defines abnormal clinical findings on chest radiograph with features that cannot be seen on chest radiograph but are found on clinical examination

			aged <5 years and 5–15 years in Chiba City was 35 885 and 92 281, respectively, in September 2018									Bacterial pneumonia: positive blood culture or the isolation of microorganisms from washed sputum samples.	
Petousis-Harris 2019 [31]	New Zealand	Retrospective national cohort study	All NZ children less than 6 years of age between 1 Jan 2006 and 31 Dec 2015 Total 344 020 and 375 720 children < 6 years of age	Nationwide	26,589	< 6 years	PCV10 & PCV13, 3+1, no	PCV7 in June 2008 PCV10 in 2011 PCV13 in 2014.	Pre-PCV: 2006 Post-PCV: 2015	4 post pCV10 1 post PCV13	83% in 2015	All-cause pneumonia (ACP): codes J12-J18, J10.0, and J11.0 (ICD-10-AM)	National Health Index (NHI) Database National Minimum Data Set (NMDS) is a national collection of public and private hospital

			between 2006-2013										discharge information
van Deursen 2017 [36]	The Netherlands	Retrospective population-based cohort study; time series	All ages in Netherlands	Nationwide [6]	155, 994 *all ages	All ages, including children <12 months, between 2-4 years and 5-17 years	PCV10, 3+1 (until Nov 2013) 2+1 (Nov 2013 onwards), not stated	PCV7 June 2006 PCV10 replaced in March 2011	Pre-PCV: Jan 1999–June 2006 Post-PCV: July 2006–Dec 2014	3.5	94%	CAP hospitalization: defined as (1) a primary discharge diagnosis of all-cause pneumonia or (2) meningitis, septicaemia or empyema as primary discharge diagnosis and pneumonia as secondary (ICD-9and ICD-10) codes)	National Medical Registration database
Vestjens 2019 [43]	The Netherlands	Retrospective laboratory-based cohort; Pre-post-comparison	All ages	National IPD surveillance in the Netherlands (25%	Pneumonia: 78 Empyema: 6	All ages, including children < 15 years	PCV10, 3+1 (until Nov 2013) 2+1 (Nov 2013	PCV7 June 2006 PCV10 replaced in	Pre-PCV: June 01 2004 - May 31, 2006 Post-PCV7: June 01 - 2008 to	5	PCV coverage in children at age 2 years has been 93–95% since the	Invasive pneumonia without empyema (detailed cause definition not included) Invasive pneumonia with empyema	Netherlands Reference Laboratory for Bacterial Meningitis

[6] Only data from hospitals that provided data for the entire study period from 1999-2014 were used, representing 38% of the total number of hospitalizations

				of the Dutch population)			onwards), not stated	March 2011	May 31, 2011<br><br>Post-PCV10: June 01, 2013 - May 31, 2016		introduction of PCV7	detailed cause definition not included)<br><br>Death: deceased during admission or within 30 days after obtaining the S. pneumoniae culture-positive material	Clinical information collected retrospectively from hospital medical records using a standardised form
Ben-Shimol 2017[7] [16]	Southern Israel	Prospective population based cohort study	30,000 children under 2 years old in 2012 in NEGEV REGION/district	Soroka University Medical Center (SUMC), only hospital in the Negev district proviing promary and referral	Pneumonia: 4,383<br><br>Non-alveolar LRTI: 116, 321	< 2 years	PCV13, 2+1, no	PCV7 July 2009<br><br>PCV 13 replacement 2010	April 2006-March 2014<br><br>Pre-PCV: April 2006-March 2009	3.5	2010: 3%<br>2011: 30%<br>2012: 86%<br>2012: 89%<br><br>In 2-11 month olds	Radiologically confirmed pneumonia (WHO-defined)<br>Non-alveolar LRTI (ICD-9)	Soroka University Medical Centre (SUMC)

[7] Case number totals include both inpatients and ED visits

				healh serviices to the entire pop. Of the region					Post-PCV: April 2013-March 201				
Jimenez-Trujillo 2017 [25]	Spain	Retrospective population-based cohort study - time-trend analysis	Spanish Children < 18 years	Nationwide	194, 419	< 18 years	PCV10 & PCV13, 2+1, not stated	PCV10 in 2009 followed by PCV13 PCV13 in 2010	Pre-PCV: mid 2001-2002<br><br>Post-PCV introduction, 7 periods:<br><br>2002-2003<br>2004-2006<br>2007-2008<br>2009<br>2010-2011<br>2012-2014	4	Children < 2 years :<br>PCV coverage<br>2010: 76%<br>2011: 66%<br>2012: 53%<br>2013: 59%<br>2014: 68%<br><br>> 55% since 2006<br><br>Children 2-4 years:<br>2010: 70%<br>2011: 67%	Community acquired pneumonia (CAP) ICD-9CM, codes: 480-488, 507.0-507.8)<br><br>In hospital mortality (IHM): is defined by the proportion of patients who died during admission for each year of study is expressed per 1000 hospitalizations	Discharge Data from Spanish National Hospital Database

											2012: 61%<br>2013: 56%<br>2014: 61%<br><br>> 55% since 2006		
Naucler 2019<br>[29]	Sweden	Retrospective population-based cohort study - Pre- post comparison	All ages in Sweden<br><br>All episodes with patients hospitalized<br>due to pneumonia in Sweden from 2005 to 15.	Nationwide	303, 691	All ages, including children < 4 years and between 5-17 years	PCV10 or PCV13<br>depending on the county, 2+1, no	PCV7 in 2007<br><br>PCV10 or PCV13 from 2009 depending on the county	Pre-PCV:<br>2005-2006<br><br>Post-PCV period:<br>2014-2015	5	Vaccine coverage for 3 doses among 2-year old<br>Children:<br>2010: 60%<br><br>2011-2017: 96.5%-97.6%	All-cause pneumonia (AC-CAP):<br>first-listed discharge diagnosis of pneumonia, or first-listed diagnosis of meningitis, septicaemia or empyema in addition to a pneumonia Diagnosis<br><br>Pneumococcal pneumonia or Lobar pneumonia (unspecified)(PL-pneumonia): ICD-10	National<br>Patient Register (NPR) at the National Board of<br>Health and Welfare

												code J13 or J18.1 respectively	
Wiese 2016 [37]	United States of America	Retrospective hospital-based cohort study; Pre-post comparison	Tennessee residents <18 years of age	All non-federal hospitals in Tennessee for 1998–2013	Not stated	< 18 years	PCV13, 3+1, no	PCV7 in 2000<br><br>PCV13 in 2010	Pre-PCV: 1998–1999<br><br>Early PCV7: 2001–2005<br><br>Late PCV7: 2006–2009<br><br>Post-PCV13: 2011–2013	3	Not stated	Pneumonia (ICD-9-CM)	Tennessee Hospital Discharge Data system
Wiese 2016 [48]	United States of America	Retrospective population based ecological cohort study	U.S children < 18 years	Nationwide	8,903	< 18 years of	PCV13, 3+1, no	PCV7 2000<br><br>PCV13 replacement 2010	Pre-PCV7: 1997–1999<br><br>Early-PCV7: 2001–2005<br><br>Late-PCV7: 2006–2009	3	≥3 doses of PCV7 was >90% in 2007–2008 and has been >92% for ≥3 doses of	Parapneumonic empyema (PE): any pneumonia (ICD9-CM: 480.0–486.9 or 510) hospitalization with a diagnosis of empyema (primary or otherwise)	Nationwide Inpatient Sample and Census data

									Post-PCV13:2011–2013		PCV13 since 2010	Pneumococcal parapneumonic empyema (PPE): parapneumonic empyema definition as above with associated discharge code: pneumococcal (ICD9-CM: 481)	

Table S3. Summary of results for all-cause pneumonia

Author	Country	Analytical Method	Adjustment Factors	Incidence Rates		Vaccine Impact Results		
				Pre-PCV	Post-PCV	Age Group	Percent Reduction (%)(95% CI), p-value (if provided)	IRR (95% CI) (if provided)
Andrade 2017 [13]	Brazil	Time-series analysis	Excluded hospitalization occurring during H1N1 influenza pandemic months in Brazil (April-October 2009), adjusted for changes in all-cause hospital admissions	Annual rates /100,000 population of pneumonia:<br><12 months<br>2005: 4335.5<br>2006: 4489.8<br>2007: 4378.4<br>2008: 4260.6<br>2009: 4741.2<br><br>12-23 months:<br>2005: 3043.4<br>2006: 3151.4<br>2007: 3098.6	Annual rates /100,000 population of pneumonia:<br><12 months<br>2011: 4067.9<br>2012: 3864.2<br>2013: 2879.8<br>2014: 2589.7<br>2015: 2433.0<br><br>12-23 months:<br>2011: 2568.5<br>2012: 2435.2<br>2013: 2389.1	<12 months	26.5 (17.5-35.5), 0.001	
						12-23 months	17.4 (8.9-25.8), 0.006	
						2-4 years	21.5 (13.2-29.8), 0.002	
						5-9 years	16.8(8.4-25.1), 0.006	

				2008: 2933.3<br>2009: 3159.4<br><br>2-4 years:<br>2005: 1120.1<br>2006: 1168.8<br>2007: 1182.8<br>2008: 1106.1<br>2009: 1219.1<br><br>5-9y ears:<br>2005: 436.7<br>2006: 370.3<br>2007: 347.4<br>2008: 339.2<br>2009: 389.3	2014: 2284.2<br>2015: 2175.1<br><br>2-4 years:<br>2011: 1066.00<br>2012: 973.7<br>2013: 955.5<br>2014: 904.0<br>2015: 834.5<br><br>5-9 years:<br>2011: 302.8<br>2012: 281.4<br>2013: 291.3<br>2014: 263.7<br>2015: 231.7			
Becker -Dreps 2017	Nicaragua	Adjusted IRR and 95% CI were estimated using	Controlled for municipality to account for distance to patients	Incidence Rate of Pneumonia	Incidence Rate of Pneumonia	< 12months:	30	0.70 (0.66,0.75)

[15]		GEE for vaccine period compared to pre-vaccine period	home municipality to the hospital and potential differences in care seeking and immunization coverage by municipality category	Hospitalizations (1,000 person-years)<br><br><12 months<br>Pre-PCV: 1,575 (64.3)<br><br>12-23 months<br>Pre-PCV: 600(24.8)<br><br>25-59 months<br>Pre-PCV: 509(7.2)<br><br>5-14 years<br>Pre-PCV: 191(0.8)	Hospitalizations (1,000 person-years)<br><br>< 12months<br>Post-PCV: 1,743 (45.4)<br><br>12-23 months<br>Post-PCV: 888(22.8)<br><br>25-59 months<br>Post-PCV: 806(6.9)<br><br>5-14 years<br>Post-PCV: 310(0.8)	12-23 months<br><br>24-59 months<br><br>5-14 years	8<br><br>5<br><br>N/A	0.92 (0.85,0.99)<br><br>0.95 (0.82,1.10)<br><br>1.05 (0.97,1.14)
de Oliveira 2020[8]	Argentina, Brazil, Colombia,	Estimated rate ratios were calculated by dividing the	None	Not stated[9]		2-59 months	Argentina: 8<br>Brazil: 2<br>Colombia: 24	Argentina: 0.92 (0.74, 1.11)<br>Brazil: 0.98 (0.92, 1.04)

[8] IRR given with 95% CrI not CI

[9] Only provides incidence rate values on graphs, does not provide incidence rate values

[21]	Ecuador, Honduras, Mexico, Nicaragua	cumulative number of observed pneumonia deaths by the cumulative number of predicted pneumonia deaths during the evaluation period				Ecuador: 25<br>Honduras: N/A<br>Mexico: 11<br>Nicaragua: 19	Colombia: 0.76 (0.65, 0.97)<br>Ecuador: 0.75 (0.59, 0.96)<br>Honduras: 1.16 (0.77, 1.5)<br>Mexico: 0.89 (0.82, 0.97)<br>Nicaragua: 0.81 (0.67, 1)
					2-11 months	Argentina: 16<br>Brazil: 8<br>Colombia: 14<br>Ecuador: 3<br>Honduras: 3<br>Mexico: 22<br>Nicaragua: 2	Argentina: 0.84 (0.72-0.99)<br>Brazil: 0.92 (0.86-0.98)<br>Colombia: 0.86 (0.65-1.05)<br>Ecuador: 0.97 (0.73-1.30)<br>Honduras: 0.97 (0.55-1.33)<br>Mexico: 0.78 (0.73-0.85)<br>Nicaragua: 0.98 (0.8-1.20)

					12-23 months	Argentina: 35<br>Brazil: 9<br>Colombia: 40<br>Ecuador: 27<br>Mexico: 3	Argentina: 0.65 (0.50-0.85)<br>Brazil: 0.91 (0.81-1.03)<br>Colombia: 0.60 (0.52-0.73)<br>Ecuador: 0.73 (0.57-0.91)<br>Mexico: 0.97 (0.85-1.11)
					24-59 months	Argentina: 1<br>Brazil: 7<br>Colombia: 10<br>Ecuador: 52<br>Mexico: N/A<br>Nicaragua: 28	Argentina: 0.99 (0.80- 1.27)<br>Brazil: 0.93 (0.75-1.14<br>Colombia: 0.90 (0.64-1.27)<br>Ecuador: 0.58 (0.29-1.10)<br>Mexico: 1.12 (0.99-1.26)

								Nicaragua: 0.72 (0.46-1.03)
Dondo 2019 [22]	Zimbabwe	Time series using a negative binomial segmented regression model was used to calculate the annual percent change in pneumonia during the pre- and post-PCV13 introduction periods. And the change in slope pre- and post-PCV13 introduction to determine if there was a statistically significant (P = .05) change in the time period after vaccine introduction	Adjusted for all cause pneumonia admissions and seasonality			0-11 months	Annual % decline<br>Pre-PCV: 11(4-17)<br>Post-PCV: 9(4-13)<br>No significant change post vs pre	
						12-59 months	Annual % decline<br>Pre-PCV: 7(0-14)<br>Post-PCV: 10 (6-15)<br>No significant change post vs pre	

Izu 2017[10] [24]	South Africa	Time series using Bayesian generalized seasonal autoregressive integrated moving-average models	Adjusted for the proportion of HIV-infected children on antiretroviral therapy (ART), the influenza season and the number of bronchiolitis-associated admissions and the transition period between PCV7 use and PCV13 use	Pneumonia hospitalizations per 1000 children with **any HIV status**<br><br>< 3 months<br>2006: 46 (43 to 49)<br>2007: 39 (36 to 42)<br>2008: 50 (47 to 53)<br><br>3-11 months<br>2006: 58 (55 to 62)<br>2007: 44 (41 to 47)<br>2008:55 (51 to 58)<br><br>1-23 months<br>2006: 29 (27 to 31)<br>2007: 23 (21 to 25)	Pneumonia hospitalizations per 1000 children with **any HIV status**<br><br>< 3 months<br>2012: 36 (34 to 38)<br>2013:38 (36 to 41)<br>2014: 28 (26 to 30)<br><br>3-11 months<br>2012: 31 (28 to 33)<br>2013: 27 (25 to 29)<br>2014: 22 (20 to 24)<br><br>1-23 months<br>2012: 18 (16 to 19)<br>2013: 15 (14 to 17)<br>2014: 14 (13 to 16)	<5 years without confirmed HIV infection	2013: 27 (13 to 39)<br>2014: 9 (24 to 50)	
						<5 years with confirmed HIV infection	2014: 33 (6 to 52)	
						< 3 months old with confirmed HIV infection	2013: 55 (27 to 72)	
						2- 59 months old with confirmed HIV infection	2013: 51 (3 to 71<br>2014: no sig. difference	

[10] Calculates % reduction with 50% CrI, not 95% CI

				2008: 26 (24 to 28)<br><br>2-59 months:<br>2006: 9 (8 to 10)<br>2007: 7 (6 to 7)<br>2008: 8 (7 to 9)<br><br><5 years<br>20006: 31 (30 to 32)<br>2007: 24 (23 to 25)<br>2008: 29 (28 to 30)	2-59 months:<br>2012: 6 (6 to 7)<br>2013: 4 (4 to 5)<br>2014: 5 (4 to 5)<br><br><5 years<br>2012: 20 (20 to 21)<br>2013: 19 (18 to 20)<br>2014: 16 (15 to17)			
Saxena 2015 [32]	England	Interrupted time series analysis	Adjusted for child's sex, seasonality and influenza like illness	All-cause pneumonia annual hospitalization incidence rate (95% CI) per 100,000 children<br><br>2001: 120.2 (118.0-122.3)<br>2002: 121.7 (119.5-123.9)<br>2003: 125.1 (122.9-127.3)<br>2004: 134.7 (132.4-137.0)<br>2005: 158.6 (156.2-161.1)	All-cause pneumonia annual hospitalisation incidence rate (95% CI) per 100,000 children<br><br>2010: 138.2 (136.0-140.5)<br>2011: 125.5 (123.3-127.6)<br>2012: 127.6 (125.5-129.7)<br>2013: 102.2 (100.3-104.1)	Doesn't report IRR or % decline comparing Pre-PCV vs. PCV13		

				2006: 141.7 (139.4-144.0)<br>2007: 122.2 (120.1-124.4)<br>2008: 121.2 (119.1-123.3)<br>2009: 131.8 (129.6-134.0)				
Mpabalwani 2019<br>**[28]**	Zambia	Monthly case counts during the pre-PCV10 period were used in a negative binomial regression models to calculated predicted case counts had PCV not been introduced.<br><br>The decline in hospitalisations or deaths was<br>calculated as the ratio of the observed to the expected data in the post-PCV10 period	Adjusted for seasonal pattern of the case counts and UTH pneumonia models, included monthly hospitalisation counts for the control conditions and an interaction term between the control hospitalization counts and the continuous time indicator.	Not stated	Not stated	<1 year	MOH DATA<br>37.8 (21.4–50.3)<br>UTH DATA: no sig. changes	
						1-4 years	MOH DATA<br>28.8% (17.7–38.7)<br>UTH DATA: no sig. changes	

		Chi-square test was used to compare case fatality ratios between pre- and post-PCV10 periods by age group						
Petous is-Harris 2019 [31]	New Zealand	Linear trends were tested using Cochrane-Armitage trend tests for changes over time, Percentage change was calculated as the difference between number of hospitalizations between 2015 and 2006	No adjustments, analysis was stratified for different ethnic groups	Incidence rates (per 100,000 person-years) (95% CI) of ACP Hospitalizations<br><br>2006: 976 (943, 1009)<br>2007: 881 (850, 913)<br>2008: 945 (913, 977)<br>2009: 981 (949, 1013)<br>2010: 801 (772, 831)	Incidence rates (per 100,000 person-years) (95% CI) of ACP hospitalisations<br><br>2011: 836 (806, 866)<br>2012: 774 (746, 802)<br>2013 : 705 (678, 732)<br>2014: 731 (704, 758)<br>2015: 801 (773, 830)<br><br>Incidence Rates (per 100,000 person-years) (95% CI) of ACP Hospitalizations 2006-2015 by age group	All children < 6 years	8	
						Maori children < 6 years	12	
						Pacific children < 6 years	21	

					< 1 year: 1713 (1680, 1746)<br>1 year: 1495 (1464, 1526)<br>2 years: 758 (736, 780)<br>3 years: 504 (487, 522)<br>4 years: 351 (336, 366)<br>5 years: 256 (243, 269)			
Anderson 2017 [12]	Canada	Cochran-Armitage tests, assessing trends in proportions. Poisson regression to test yearly frequency rates. Compared 2000-2001 to 2013-2014	Transfers or readmissions within 7 days or less after previous discharge date were considered one hospitalisation episode	Not stated[11]		6-23 months	18.2	
						24-59 months	22.5	
Andriatahirintsoa 2019 [14]	Madagascar	Comparison of pneumonia hospitalisations before and after PCV10 introduction represented as %s	None	% of all hospitalisations due to pneumonia Pre-PCV period: 24.5%<br><br>% of total hospitalisation due to pneumonia:	% of all hospitalisations due to pneumonia Post-PCV period: 19.0%<br><br>% of total hospitalisation due to pneumonia	<5 years	22 (p<0.001)	

[11] Only provides incidence rate values on graphs, does not provide incidence rate values

				2010: 27%<br>2011: 22%<br>2012: 29%	2013: 20%<br>2014: 15%<br>2015: 16%<br>2016: 25%<br>2017: 19%			
Congdon 2020 [19]	Botswana	A seasonally adjusted interrupted time-series analysis using negative binomial regression model was used to evaluate the effect of introduction of these vaccines on child pneumonia hospitalizations and deaths.	Adjusted for seasonal trends	Annual rate of change (95% ci) in the number of pneumonia hospitalizations<br>All ages:<br>Rate: 1.24 (CI: 0.94-1.64)<br>1-11months:<br>Rate: 1.39 (CI: 0.94,1.64)<br>12-59months:<br>Rate: 1.03 (CI: 0.77,1.37)	Annual rate of change (95% ci) in the number of pneumonia hospitalizations<br>All ages: :0.94(0.89-0.99)<br>1-11 months: 0.94(088-0.999)<br>12-59 months: 0.94(089-0.996)	1-11 months	Annual decline of 6%) during the post-vaccine period	Annual rate of decline in post-PCV period: 0.94 (0.88, 0.999)
						12-59 months	Annual decline of 6% during the post-vaccine period	Annual rate of decline in post-PCV period: 0.94 (0.89, 0.996)
						1-59 months	Annual decline of 6% during the post-vaccine period	Annual rate of decline in post-PCV period: 0.94 (0.89, 0.99)
	Brazil		None			< 1 year	23.3, p<0.005	

Kupek 2016 [26]		Time series using the Poisson regression coefficient, difference in the time trend between the vaccination periods was calculated by comparing the gradients of the annual rates for each period.		Annual pneumonia hospitalisation rate (95%CI) (per 1000 live births) < 1 year: 49.8 (49.0-50.5) 1-4 years old: 14.1 (13.9-14.3)	Annual pneumonia hospitalisation rate (95%CI) (per 1000 live births) < 1 year: 38.2 (37.6 -38.8) 1-4 years old: 13.0 (12.8-13.1)	1-4 years	8.4, p<0.005,	
Luca 2018 [27]	Canada	Difference in - Difference out analysis: using regression models to compare the before-and-after-intervention difference in outcomes for groups affected by	Accounted for potential seasonality of pneumonia and non–pneumonia-related outcomes	Not stated[12]		< 2 years	Pre-PCV vs. PCV10 37.8 (32.4–43.0), p< 0.05 Pre-PCV vs. PCV13 45.3 (39.8-50.7), p< 0.05	

[12] Only provides incidence rate values on graphs, does not provide incidence rate values

		the intervention with the difference for unaffected groups.				5-17 years	Pre-PCV vs. PCV10<br>25.6 (33.7-17.4), p< 0.05<br><br>Pre-PCV vs. PCV13<br>80.7 (87.2-74.2), p< 0.05	
Wiese 2016 [37]	United States of America	For comparisons, rate ratios and rate differences were calculated with 95% CIs to assess significance of the estimates at the 5% level	None	Annual pneumonia hospitalizations (per 1,000 children)<br><br>< 18 years: 4.0<br>< 2 years: 18.4<br>2-4 years: 18.4<br>5-17 years: 1.6	Annual pneumonia hospitalizations (per 1,000 children)<br><br>< 18 years: 2.0<br>< 2 years: 7.3<br>2-4 years: 3.0<br>5-17 years: 1.1	< 2 years	60	0.40 (0.38–0.41)
						2-4 years	44	0.56 (0.52–0.59)
						5-17 years	33	0.67 (0.63–0.70)
						< 18 years	49	0.51 (0.50–0.52)

Sigurdsson 2020 [34]	Iceland	Crude incidence rate ratios (IRR) were calculated between the VNEC and VEC assuming Poisson variance	None	Incidence rate (per 1,000 person-years) for pneumonia hospitalization in VNEC: 4.94	Incidence rate (per 1,000 person-years) for pneumonia hospitalization in VEC: 4.18	12-17 months	48	0.52 (0.35–0.77)
						< 3 years	20	0.80
Alvarado 2018 [11]	Chile	IRR + 95% CI estimated using Poisson regression model, using 2010 as reference year (pre-PCV) to compare to period 2011-2015	Not described	CAP incidence rate (per 10,000 children <5yrs)<br>2009: 46.7<br>2010: 59.3	CAP incidence rate (per 10,000 children <5yrs)<br>2011: 55<br>2012: 49.7<br>2013: 45.1<br>2014: 42.9<br>2015: 34	<5 years	8, p < 0.001	0.92 (0.91-0.93)
da Silva 2016 [20]	Brazil	a multivariate logistic regression analysis was performed and all explanatory variables were included in the model (number of paediatric CAP hospitalizations,	Age, sex, municipality of residence, vaccination status and vaccination coverage	Rates not presented, only number of cases	Rates not presented, only number of cases	< 1 year	19, p < 0.05	0.81 (0.74-0.89)

		adjusted age, sex, municipality of residence, vaccination status, vaccination coverage)						
van Deursen 2017 [36]	The Netherlands	Time series analysis using Poisson regression comparing the observed time trend after introduction of PCV with the predicted linear time trend	Rates not presented	Rates not presented	Time series adjusts for secular trends, no other adjustments described	0-6 months	38	0.62 (0.41–0.96)
						6-12 months	33	0.67 (0.50–0.90)
						2-4 years	22	0.78 (0.61–0.97)
						5-17 years	22, not significant	0.88 (0.63–1.23)
Jimenez-Trujillo 2017 [25]	Spain	Incidence rates per 100,000 inhabitants calculated: no. of admissions per year / no. of people in that population group according to	Poisson regression, adjusted by age and sex when needed, and the year 2009was analysed separately - year of H1N1 influenza pandemic	Incidence of hospital admissions for CAP (per 100,000 children) PRE-PCV10/13<br><br>< 2 years<br>2001: 601.67 2002: 635.74 2003: 620.88 2004: 583.6 2005: 603.01	Incidence of hospital admissions for CAP (per 100,000 children)<br><br>< 2 years<br>2009: 658.73 2010: 478.35 2011: 486.2 2012: 392.97 2013: 346.44 2014: 396.57	< 2 years	% Annual Reduction: 3.67 *significant decrease 2001-2014	

		census data. Linear joint point regression to identify trend changes in CAP incidence rates, then a time-trend analysis was performed using Poisson regression		2006: 595.48 2007: 576.11 2008: 541.4<br><br>2-4 years:<br>2001: 431.71 2002: 421.33 2003: 452.84 2004: 398.12 2005: 419.41 2006: 478.56 2007: 469.9 2008: 398.54<br><br>5-9 years:<br>2001: 137.83 2002: 90.17 2003: 110.58 2004: 108.54 2005: 118.99 2006: 156.57 2007: 162.85 2008: 118.89	2-4 years:<br>2009: 480.68 2010: 372.59 2011: 383.54 2012: 246.98 2013: 255.97<br>2014: 280.22<br><br>5-9 years:<br>2009: 161.03 2010: 113.94 2011: 123.16 2012: 70.01 2013: 85.21 2014: 93.95	2-4 years	% Annual Reduction: 11.4<br>* only significant decrease from 2010-2014	
						5-9 years	No significant changes 2001-2014	
Faye 2019<br>[23]	Senegal	Poisson regression to compare hospitalizations before and after PCV introduction<br><br>Calculated proportions of hospitalizations with discharge	Adjusted for seasonality<br><br>Transitional period was excluded from analysis	Average pneumonia hospitalization (% of total all-cause hospitalizations)<br><br><12 months: 7.9%<br>12-59 months: 17.7%<br>0-59 months: 12.8%	Average pneumonia hospitalization (% of total all-cause hospitalizations)<br><br><12 months: 6.5%<br>12-59 months: 14.6%<br>0-59 months: 10.1 %	<12 months	3.8 (1.5-5.9)	
						12-59 months	0.7% (-0.8-2.2%)<br>* not statistically significant	

		diagnosis of pneumonia						
Sgambatti 2016 [33]	Brazil	Linear regression using Poisson distribution to assess the rate change in the post-PCV period compared to the pre-PCV period	Adjusted for secular trends	Clinical Pneumonia hospitalization incidence (per 100,000 children) (95% CI)<br><br>2–23 months: 5728 (5548–5912)<br>2–11 months: 6788 (6505–7081)<br>12–23 months: 4802 (4577–5035)<br>24–35 months: 2408 (2248–2576)<br>Total: 4565 (4436–4699	Clinical Pneumonia hospitalization incidence (per 100,000 children) (95% CI)<br><br>2–23 months: 4976 (4806–5151)<br>2–11 months: 5935 (5667–6211)<br>12–23 months: 4122 (3911–4344)<br>24–35 months2229 (2073–2394)<br>Total: 4025 (3901–4153)	2-11 months	12.6 (12.3–12.9)	
						2-23 months	13.1 (12.9–13.4)	
						12-23 months	14.2 (13.7–14.6)	
						24-35 months	7.4 (7.1–7.8)	
						2-35 months	11.8 (11.6–12.1)	
Kabore 2020 **[39]**	Burkina Faso	Pre and Post PCV trends were generated with segmented regression analysis.	Adjusted for seasonality, pre-PCV trends and a binary variable for a free care policy implemented nationwide in 2017	Incidence rates not stated but the pre-PCV trend is described as increasing by 2% per month	Incidence rates not stated no trend observed post-PCV	0-59 months	Change in intercept: 34, p = 0.001<br>Change in slope: 3.2, p <0.001	Change in intercept: 0.66 (0.51; 0.84)<br>Change in slope: 0.968 (0.955; 0.982)

		Calculated IRR and incidence rates				0-23 months	Change in intercept: 24, p = 0.03<br>Change in slope: 4.1, p <0.001	Change in intercept: 0.76 (0.59; 0.98)<br>Change in slope: 0.959 (0.945; 0.973)
						24-59 months	Change in intercept: 50, p <0.001<br>Change in slope: 1.6, p= 0.08, non-significant	Change in intercept: 0.50 (0.36; 0.70)<br>Change in slope: 0.984 (0.967; 1.002)
Berger 2019 [17]	Israel	Simple linear regression analysis used to determine trends in hospitalization rates and both annual and period % changes were calculated	N/A	LRTI Hospitalization rates/ 100,000 = 142.1	LRTI Hospitalization rates / 100,000 = 80.9	< 18 years	43, p < 0.0001	

Ben-Shimol 2017 [16]	Southern Israel	IRR and 95% CI calculated comparing 3 years between April 2006 and March 2009 (pre-PCV) with the last study year (April 2013 - March 2014)	Accounted for H1NI influenza outbreak in April 2009-March 2010	Annual rates per 1000 children < 2 years of age: Non-alveolar LRTI<br>2006-2007: 34.6<br>2007-2008: 30.5<br>2008-2009: 32.3<br>2009-2010: 72.3	Annual rates per 1000 children < 2 years of age: Non-alveolar LRTI<br>2006-2007: 42.7<br>2007-2008: 30.8<br>2008-2009: 31.4<br>2009-2010: 27.9	< 2 years	7	0.93 (0.87-0.99)
Naucler 2019 [29]	Sweden	Poisson regression was used to calculate incidence rate ratios (IRR) with 95% confidence intervals (CI to compare pneumonia in 2014–15 (post-vaccination period)	Adjusted for trends in admittance practices using four control conditions. Age adjusted incidence rates to year 2005	Incidence of all cause pneumonia Pre-PCV per 100,000 person-years:<br>< 2 years: 654<br>2-4 years: 251<br>5-17 years: 49	Incidence of all cause pneumonia Post-PCV per 100,000 person-years:<br>< 2 years: 417<br>2-4 years: 201<br>5-17 years: 4	< 2 years	36 (32–40)	0.64 (0.60–0.68)
						2-4 years	20 (14–25)	0.80 (0.75–0.86)
						5-17 years	16 (11–22)	0.84 (0.78–0.91)
				Incidence of PL-pneumonia[13]	Incidence of PL-pneumonia	< 2 years	58 (35–72)	0.42 (0.28–0.65)

[13] PL-pneumonia: pneumococcal pneumonia or lobar pneumonia

		with 2005–06 (pre-vaccination period).		Pre-PCV per 100,000 person-years:<br><br>< 2 years: 17.5<br>2-4 years: 8.7<br>5-17 years: 2.9	Post-PCV per 100,000 person-years:<br><br>< 2 years: 7.4<br>2-4 years: 4.3<br>5-17 years: 1.1	2-4 years	51 (23–69)	0.49 (0.31–0.77)
						5-17 years	64 (45–76)	0.36 (0.24–0.55)
Palmu 2017 **[30]**	Finland	Pneumonia rates in the target cohort were compared to rates in the combined reference cohorts using Poisson regression models. Relative rate reduction (percent) was calculated as (1 ±relative risk) 100%.	To reduce the effect seasonal variation on annual estimates yearly rates were calculated by epidemic years (July to June).	Incidence rate (per 1,000 person-years) HDP<br><br>Direct Target Cohort<br>Pre-PCV1<br>= 10.3<br><br>Indirect Target Cohort<br>Pre-PCV2<br>= 6.3	Incidence rate per 1,000 person-years) HDP<br><br>Direct Target Cohort<br>Post-PCV1<br>= 9.0<br><br>Indirect Target Cohort<br>Post-PCV2<br>= 6.3	3-24 months	13 (9-16)	
						7-71 months	-1 (-7, 5)	
				Incidence rate (per 1,000 person-years) HTP<br><br>Direct Target Cohort	Incidence rate per 1,000 person-years) HTP<br><br>Direct Target Cohort	3-24 months	23 (18-28)	
						7-71 months	18 (10, 25)	

				Pre-PCV1<br>= 5.35<br><br>Indirect Target Cohort<br>Pre-PCV2<br>= 3.2	Post-PCV1<br>= 4.1<br><br>Indirect Target Cohort<br>Post-PCV2<br>= 2.6			
Thorrington 2018[14]<br>**[35]**	England	Composite Control Method	To account for biases arising from potential secular<br>trends in admission practice over the study period, the<br>IRRs of each disease endpoint were compared to the<br>IRRs of five control conditions that should not be affected<br>by changes in the introduction of the PCV	Not stated[15]		< 2 years	34	0.66 [0.51, 0.89]
						2-4 years	20	0.80 [0.62, 1.11]
						5-14 years	33	0.67 [0.59, 0.87]

[14] Adjusted IRR(rIRR) are calculated with [min-max] not 95% CI

[15] Only provides incidence rate values on graphs, does not provide incidence rate values

			programme				

Table S4. Summary of results for Severe Pneumonia								
**Author**	**Country**	**Analytical Method**	**Adjustment factors**	**Incidence Rates**		**Vaccine Impact Results**		
				**Pre-PCV**	**Post-PCV**	**Age Group**	**Percent Reduction (%)(95% CI), p-value (if provided)**	**IRR (95% CI) (if provided)**
Kabore 2020 [39]	Burkina Faso	Pre and Post PCV trends were generated with segmented regression analysis. Calculated IRR and incidence rates	Adjusted for seasonality, pre-PCV trends and a binary variable for a free care policy implemented nationwide in 2017	Incidence rates not stated	Incidence rates not stated	0-59 months	Change in intercept: 36, p=0.001<br>Change in slope: 3, p <0.001	Change in intercept: 0.64 (0.49; 0.84)<br>Change in slope: 0.970 (0.956; 0.984)
						0-23 months	Change in intercept: 26, p=0.04<br>Change in slope: 4.1, p <0.001	Change in intercept: 0.74 (0.56; 0.98)<br>Change in slope: 0.959 (0.944; 0.974)
						24-59 months	Change in intercept: 55, p <0.001<br>Change in slope: 0.4, p=0.74, not significant	Change in intercept: 0.45 (0.30; 0.68)<br>Change in slope: 0.996 (0.975; 1.018)
	Kenya				Not stated	2-59 months	*Severe: 40,p=0·017	*Severe: 0·60 (0·40–0·91)

Silaba 2019 [42]		Interrupted Time series analysis by fitting a linear regression models to log-transformed monthly rates of pneumonia. The models included a period effect (pre-PCV10 vs postPCV10), monthly time trend, and seasonality, which was modelled using the month of the year. Differences in the time trends before and after vaccination were tested through the inclusion of an	Adjusted for seasonal and temporal trends	Clinically pneumonia - severe or very severe incidence per 100,000 person-years<br><br>2002-2003:<br>2-59 months: 2170<br><br>2010: 1220			V sever: 13, p=0·519<br>*All pneumonia: 27, p=0.033	V sever: 0·87 (0·56–1·34)<br>*All pneumonia: 0·73 (0·54–0·97) 0.033, 27%
						2-11 months	Severe: 34, p=0·090<br>V severe: 27, p=0·143<br>*All pneumonia: 030, p= 0.048	Severe: 0·66 (0·41–1·07)<br>V severe: 0·73 (0·48–1·11)<br>*All pneumonia: 0·70 (0·50–1·00)
						12-23 months	*Severe: 39, p=0·027<br>V severe: N/A, p=0·264<br>All pneumonia: 16, p=0.283	*Severe: 0·61 (0·39–0·94)<br>V severe: 1·28 (0·83–1·96)<br>All pneumonia: 0·84 (0·61–1·15)
						24-59 months	*Severe: 41, p=0·020<br>V severe: 22, p=0·479<br>All pneumonia: 19, p=0·192	*Severe: 0·59 (0·37–0·92)<br>V severe: 0·78 (0·40–1·54)<br>All pneumonia: 0·71 (0·43–1·19)
						60-143 months	Severe: 7, p=0·721<br>V severe: 4, p=0·857	Severe: 0·93(0·62–1·39)<br>V severe: 0·96 (0·61–1·50)

		interaction term.					All pneumonia 5, p=0·832	All pneumonia 0·95 (0·56–1·59)
						*statistically significant		
Haji 2018<br>[40]	Canada	Trends were analyzed using linear regression or one-way analysis of variance (ANOVA). Proportions were compared using Chi square or Fisher exact tests	None	Incidence COMP per 100,000 children<br><br>< 18 years:<br>2002: 3.932<br>2003: 5.732<br>2004: 3.603<br><br>< 4 years:<br>2002: 9.650<br>2003: 9.673<br>2004: 8.107<br><br>5 years - < 18 years:<br>2002: 2.298<br>2003: 4.606	Incidence COMP per 100,000 children<br><br>< 18 years:<br>2012: 12.502<br>2013: 7.024<br>2014: 12.972<br>2015: 14.120<br><br>< 4 years:<br>2012: 16.547<br>2013: 14.973<br>2014: 16.678<br>2015: 13.377	*IRR or % change not reported* only reports Chi-Square Values		
						< 4 years	Chi square = 3.23 p value 0.072<br>*no significant changes	
						5 years - < 18 years	Chi square =28.30 p value < 0.001<br>*no decrease in incidence	
						<18 years	Chi square = 29.00, p value < 0.001<br>* no decrease in incidence	

				2004: 2.316	5 years - < 18 years:<br>2012: 11.347<br>2013: 4.753<br>2014: 11.913<br>2015: 14.332			
Vestjens 2019 [16]<br>[43]	The Netherlands	Differences in incidences and proportions between<br>the time periods were tested with v2 tests and RRs with 95% CI<br>were calculated. (No further details are stated)	If considered appropriate, multivariable<br>logistic regression analyses were performed to adjust for<br>age and/or comorbidities when assessing associations between<br>time periods and outcomes. (No further details stated)	Incidence/ 100,000<br>Invasive Pneumonia without empyema<br><5 years: 4.38<br>5-17 years: 0.70	Incidence/ 100,000<br>Invasive Pneumonia without empyema<br><5 years: 0.45<br>5-17 years: 0.63	<5 years	90	0.10 (0.03-0.35)
						5-17 years	10	0.90 (0.38-2.15)

[16] Uses the term “relative risk” in result calculation, but appears to be equal to a rate ratio calculation

Mackenzie 2017 [41]	The Gambia	Poisson distribution to calculate incidence rate ratios (IRRs) and 95% CIs of the incidence in the last 2 years of surveillance (2014–15) to the incidence in the baseline first 2 years (May 12, 2008, to May 11, 2010	adjusted for observed increases in the number of children referred to clinicians per unit population over time by multiplying annual event counts by a correction factor that assumed the rate of referral in the absence of bias was constant	Adjusted Annual Incidence clinical pneumonia 1,000 person-years<br><br>2-11months: 107.4<br>12-23 months:93.3<br>2-4 years: 28.2	Adjusted Annual Incidence clinical pneumonia 1,000 person-years<br><br>2-11months: 98.1<br>12-23 months: 99.2<br>2-4 years: 30.3	2-11 months	2	0·98 (0·92–1·04)
						12-23 months	N/A	1·06 (0·98–1·15)
						2-4 years	N/A	1·07 (0·98–1·18)
						2-59 months	8 (3-13)	
				Adjusted Annual Incidence clinical hypoxemic pneumonia 1,000 person-years<br><br>2-11 months: 13.1	Adjusted Annual Incidence clinical hypoxemic pneumonia 1,000 person-years	2-11 months	57 (42-67)	0·43 (0·33–0·58)
						12-23 months	72 (58-82)	0·28 (0·18–0·42)
						2-4 years	56	0·44 (0·26–0·77)
						2-59 months	61 (52-68)	

				12-23 months: 6.8<br>2-4 years: 1.3	2-11 months: 5.7<br>12-23 months: 1.9<br>2-4 years: 0.6			
Thorrington[17] 2018<br>[35]	England	Composite Control Method	To account for biases arising from potential secular trends in admission practice over the study period, the IRRs of each disease endpoint were compared to the IRRs of five control conditions that should not be affected	Not stated[18]		< 2 years	51	0.49 [0.38, 0.65]
						2-4 years	30	0.70 [0.54, 0.98]
						5-14 years	58	0.42 [0.37, 0.55]
						No significant reduction was seen in the incidence of lung abscess with pneumonia for any age group.		

[17] Adjusted IRR (rIRR) are calculated with [min-max] not 95% CI

[18] Only provides incidence rate values on graphs, does not provide incidence rate values

			by changes in the introduction of the PCV programme		

Table S5. Summary of results for All-Cause Empyema

Author	Country	Analytical Method	Adjustment factors	Incidence Rates		Vaccine Impact Results		
				Pre-PCV	Post-PCV	Age Group	Percent Reduction (%)(95% CI), p-value (if provided)	IRR (95% CI) (if provided)
Saxena 2015 [32]	England	Interrupted time series analysis	Adjusted for child's sex, seasonality and influenza like illness	Empyema hospitalization incidence rate (95% CI) per 100,000 children<br>2001: 1.6 (1.4-1.9)<br>2002: 1.9 (1.6-2.1)<br>2003: 2.0 (1.7-2.3)<br>2004: 2.9 (2.6-3.3)<br>2005: 3.9 (3.5-4.3)	Empyema hospitalization incidence rate (95% CI) per 100,000 children<br>2010: 3.9 (3.5-4.2)<br>2011: 2.8 (2.43.1)<br>2012: 2.6 (2.3-2.9)	Doesn't report IRRs or % decline comparing Pre-PCV vs. PCV13		

				2006: 3.4 (3.0-3.7)<br>2007: 3.4 (3.0-3.8)<br>2008: 3.5 (3.2-3.9)<br>2009: 4.1 (3.7-4.5)	2013: 1.9 (1.6-2.1)			
Anderson 2017 [12]	Canada	Cochran-Armitage tests, assessing trends in proportions. Poisson regression to test yearly frequency rates. Compared 2000-2001 to 2013-2014	Transfers or readmissions within 7 days or less after previous discharge date were considered one hospitalization episode	Not stated[19]		Children 6-59months, no significant trend in incidence over time for empyema, p= 0·4		
Zampoli 2015 [49]	South Africa	Difference between IRs of 2 populations was tested using a large sample Z test. Asymptotic confidence intervals (CIs) for IR	None	Empyema incidence /1000 pneumonia admissions<br>2007: 17.9	Empyema incidence /1000 pneumonia admissions<br>2012: 4.5	<12 years	50	

[19]Only provides incidence rate values on graphs, does not provide incidence rate values

		and incidence rate ratio were con-structed using large sample theory.		2008: 8.3<br>2009: 7.4<br>2010: 11.6<br>2011: 9.5	2013: 4.4<br>2014: 3.6			
Vestjens 2019[20] [43]	The Netherland s	Differences in incidences and proportions between the time periods were tested with v2 tests and RRs with 95% CI were calculated. (No further details are stated)	If considered appropriate, multivariable logistic regression analyses were performed to adjust for age and/or comorbidities when assessing associations between time periods and outcomes. (No further details stated)	Incidence/ 100,000<br>Pneumonia with empyema<br><5 years: 0.00<br>5-17 years: 0.00	Incidence/ 100,000<br>Pneumonia with empyema<br><5 years: 0.29<br>5-17 years: 0.05	<5 years	N/A	N/A
						5-17 years	N/A	N/A
Wiese 2016 [48]	United States of American	Annualized period rates per 100,000 population were calculated by	Year of PCV introduction - 2000(pCV7(PCV13) and 2010 excluded in analysis.	Annualized rates of Parapneumonic Empyema related hospitalizations per	Annualized rates of Parapneumonic Empyema related	< 2 years	23	0.77 (0.61, 0.96)
						2-4 years	N/A, non-significant	1.02 (0.77, 1.35)

[20] Uses the term “relative risk” in result calculation, but appears to be equal to a rate ratio calculation

		dividing the weighted number of parapneumonic empyema hospitalizations within each period by the average annual population in each period, and divided by the number of years within the period. Variance estimates of the weighted number of empyema hospitalizations were used to calculate 95% confidence intervals (CI) for the annualized period rates.<br>Rates of hospitalizations for parapneumonic empyema were compared using relative rates		100,000 children by age group,<br>Pre-PCV7<br>< 2 years 4.8<br>2-4 years 3.8<br>5-17 years 1.3<br>< 18 years: 2.1 (1.7–2.4)	hospitalizations per 100,000 children by age group<br>Post- PCV13<br>< 2 years 3.7<br>2-4 years 3.9<br>5-17 years 1.3<br>< 18 years: 2.0 (1.7, 2.3)	5-17 years	N/A, non-significant	1.00 (0.79, 1.27)
						< 18 years	N/A, non-significant	0.95 (0.76–1.18)
				Annualized rates of Pneumococcal Parapneumonic Empyema related hospitalizations per 100,000 children by age group,<br>Pre-PCV7	Annualized rates of Pneumococcal Parapneumonic Empyema related hospitalizations per 100,000 children by age group	< 2 years	68	0.32 (0.28, 0.38)
						2-4 years	42	0.58 (0.46, 0.75)
						5-17 years	48	0.52 (0.44, 0.63)
						< 18 years	Not stated	Not stated

		(RR) and 95% CIs.		< 2 years 1.7<br>2-4 years 1.4<br>5-17 years 0.4	Post- PCV13<br><br>< 2 years 0.5<br>2-4 years 0.8<br>5-17 years 0.2			
Chacon-Cruz 2019<br>[47]	Mexico	Statistical analysis was purely descriptive. Impact measured by comparing cases per month before and after PCV13 implementation (may 2012)	None	Not stated	Not stated	< 16 years	Pneumococcal Empyema: 56.1<br><br>All-cause Empyema: 2.1 increase* No significant decrease	
Palmu 2017<br>[30]	Finland	Pneumonia rates in the target cohort were compared to rates in the combined reference<br>cohorts using Poisson regression models. Relative rate reduction (percent) was	To reduce the effect seasonal variation on annual estimates yearly rates<br>were calculated by epidemic years (July to June).	Incidence rate (per 1,000 person-years) Empyema<br><br>Direct Target Cohort<br>Pre-PCV1 = 0.0155	Incidence rate per 1,000 person-years) Empyema<br><br>Direct Target Cohort	3-24 months	3 (-174-70)	
						7-71 months	100 (-240, 100)	

		calculated as (1 ±relative risk) 100%.		Indirect Target Cohort<br>Pre-PCV2 = 0.01	Post-PCV1 = 0.015<br><br>Indirect Target Cohort<br>Post-PCV2 = 0.00			
Thorring ton 2018<br>[35]	England	Composite Control Method	To account for biases arising from potential secular<br>trends in admission practice over the study period, the<br>IRRs of each disease endpoint were compared to the<br>IRRs of five control conditions that should not be affected<br>by changes in the introduction of the PCV	Not stated[21]		< 2 years	56	0.44 [0.34, 0.59]
						2-4 years	43	0.57 [0.44, 0.79]
						5-14 years	57	0.43 [0.37, 0.56]

[21] Only provides incidence rate values on graphs, does not provide incidence rate values

			programme				

Table S6. Summary of results for radiologically confirmed pneumonia								
**Author**	**Country**	**Analytical Method**	**Adjustment factors**	**Incidence Rates**		**Vaccine Impact Results**		
				**Pre-PCV**	**Post-PCV**	**Age Group**	**Percent Reduction (%)(95% CI), p-value (if provided)**	**IRR (95% CI) (if provided)**
Sgambatti 2016 [33]	Brazil	Linear regression using Poisson distribution to assess the rate change in the post-PCV period compared to the pre-PCV period	Adjusted for secular trends	Radiologically confirmed pneumonia (WHO-defined) hospitalization incidence per 100,000 children (95% CI)<br><br>2–23 months: 2497 (2377–2621)<br>2–11 months: 2871 (2684–3067)<br>12–23 months 2151 (2000–2309)	Radiologically confirmed pneumonia (WHO-defined) hospitalization incidence per 100,000 children (95% CI)<br><br>2–23 months: 1862 (1757–1971)<br>2–11 months: 2142 (1981–2315)<br>12–23 months 1611 (1479–1753)<br>24–35 months 889 (792–997)	2-11 months	25.3 (24.6–26.1)	
						2-23 months	25.4 (24.7-26.0)	
						12-23 months	25.1 (24.0-26.0)	
						24-35 months	11.9 (11.3-12.7)	
						2-35 months	22.8 (22.3-24.2)	

				24–35 months 1009 (908–1121)<br>Total: 1976 (1890–2066)	Total: 1525 (1449–1605)			
Silaba 2019 [42]	Kenya	Interrupted Time series analysis by fitting a linear regression models to log-transformed monthly rates of pneumonia. The models included a period effect (pre-PCV10 vs postPCV10), monthly time trend, and seasonality, which was modelled using the month of the year. Differences in the time trends before and after vaccination were tested through the inclusion of an interaction term.	Adjusted for seasonal and temporal trends	Radiologically confirmed pneumonia (WHO-defined) incidence (95% CI) per 100,000 person-years<br>Pre-PCV:<br>2-59 months: 180.9 (163.0–200.2)	Radiologically confirmed pneumonia (WHO-defined) incidence (95% CI) per 100,000 person-years Post-PCV:<br>2-59 months: 110.7 (93.3–130.3)	2-59 months	48, p=0·011*	0·52 (0·32–0·86)
						2-11 months	27, p=0·324	0·73 (0·39–1·36)
						12-23 months	46, p=0·091	0·54 (0·27–1·10)
						24-59 months	50, p=0·052	0·50 (0·24–1·01)
						60-143 months	11, p=0·724	0·89 (0·47–1·69)
						*=statistically significant		
	Finland		None			< 36 months	40	

Laak sone n 2016 [50]		Incidences are presented as annual incidences for the years 2008, 2009, 2012 and 2013. They are also combined for the pre-vaccination period 2008–2009 and presented with their 95% confidence intervals (95% CI)		Incidence/ 10,000 children (95% CI) of hospitalization for radiologically confirmed pneumonia:<br><br>1-35 months:<br>2008: 10.8 (6.6-16.7)<br>2009: 11.8 (7.4-17.8)<br>Average Pre-PCV: 11.3 (8.1–15.3)<br><br>1-11 months:<br>2008: 1.9 (0.1-9.2)<br>2009: 5.5 (1.4-15.0)<br>Average Pre-PCV: 3.7 (1.2–9.0)<br><br>12-23 months:<br>2008: 21.1 (11.4-35.8)<br>2009: 18.8 (9.9-32.6)	Incidence/ 10,000 children (95% CI) of hospitalization for radiologically confirmed pneumonia:<br><br>1-35 months:<br>2012: 11.0 (6.8-16.8)<br>2013: 4.7 (2.2–8.8)<br><br>1-11 months:<br>2012: 7.2 (2.3-17.4)<br>2013: 3.8 (0.6–12.7)<br><br>12-23 months:<br>2012: 10.3 (4.2-21.4)<br>2013: 3.3 (0.6–10.8)<br><br>24-35 months:<br>2012: 15.3 (7.4-28.0)	1-11 months	No significant change	
						12-23 months	No significant change	
						24-35 months	No significant change	

				Average Pre-PCV: 19.9 (12.9–29.4)<br><br>24-35 months:<br>2008: 8.8 (3.2-19.5)<br>2009: 10.5 (4.3-21.8)<br>Average Pre-PCV: 9.7 (5.1–16.8)	2013: 6.8 (2.2–16.5)			
Triadou 2020 [53]	Israel	Incidence rate ratios and 95% confidence intervals (CI) were calculated for PE-CAP[22] and NPE-CAP[23]. Mean incidences during the PCV7 and PCV13 periods were compared with those of the pre-PCV period	None	Radiologically confirmed pneumonia (WHO-defined) annual incidence per 1000 children < 60 months<br>Year, PE-CAP IR, NPE-CAP IR (case no.)<br>Pre-PCV<br>Jul 2002–Jun 2003 0.20 (n.14) 16.54 (n.1135)	Radiologically confirmed pneumonia (WHO-defined) annual incidence per 1000 children < 60 months<br>Year, PE-CAP iR, NPE-CAP IR (case no.)<br>PCV13<br><br>Jul 2013–Jun 2014 0.04 (n.3) 6.52 (n.533)	< 24 months	PE-CAP: 57<br>NPE-CAP: 53	PE-CAP: 0.43 (0.21–0.85<br>NPE-CAP: 0.48 (0.45–0.51)
						24-59 months	PE-CAP: 79<br>NPE-CAP: 60	PE-CAP: 0.21 (0.09–0.46)

[22] PE-CAP: community acquired pneumonia with pleural effusion

[23] NPE-CAP: community acquired pneumonia without pleural effusion

				Jul 2003–Jun 2004 0.10 (n.7) 17.35 (n.1220)<br>Jul 2004–Jun 2005 0.18 (n.13) 13.22 (n.942)<br>Jul 2005–Jun 2006 0.21 (n.15) 14.09 (n.1011)<br>Jul 2006–Jun 2007 0.30 (n.22) 13.74 (n.995)<br>Jul 2007–Jun 2008 0.36 (n.26) 14.15 (n.1030)<br><br>PE-CAP and NPE-CAP mean<br>annual incidences per 1000 children <60 months<br>were 0.23 ± 0.09 and 14.85 ± 1.68, respectively<br><br>In the pre-PCV period, PE-CAP annual rates in children	Jul 2014–Jun 2015 0.04 (n.3) 6.37 (n.534)<br>Jul 2015–Jun 2016 0.13 (n.11) 7.32 (n.631)<br><br>PE-CAP and NPE-CAP mean<br>annual incidences not stated			NPE-CAP: 0.40 (0.36–0.44)
						<5 years	PE-CAP: 70<br>NPE-CAP: 55	PE-CAP: 0.30 (0.18–0.50)<br>NPE-CAP: 0.45 (0.43–0.48)

				<24 and 24–59 months were 0.22 ± 0.09 and 0.23 ± 0.13,<br>respectively<br><br>In the pre-PCV period, NPE-CAP annual rates in children<br><24 and 24–59 months were 24.14 ± 2.64 and<br>8.47 ± 1.05, respectively				
Ben-Shimol 2017<br>[16]	Southern Israel	IRR and 95% CI calculated comparing 3 years between April 2006 and March 2009 (pre-PCV) with the last study year (April 2013 - March 2014)	Accounted for<br>H1NI influenza outbreak in April 2009-March 2010	Radiologically confirmed pneumonia (WHO-defined) Annual rates per 1000 children < 2 years of age:<br>2006-2007: 15.8<br>2007-2008: 14.3<br>2008-2009: 16.3<br>2009-2010: 16.3	Radiologically confirmed pneumonia (WHO-defined) Annual rates per 1000 children < 2 years of age:<br><br>2010-2011: 15.3<br>2011-2012: 9.3<br>2012-2013: 9.4<br>2013-2014: 8.3	< 2 years	46, p < 0.05	0.54 (0.47-0.61)

Mackenzie 2017 [41]	The Gambia	Poisson distribution to calculate incidence rate ratios (IRRs) and 95% CIs of the incidence in the last 2 years of surveillance (2014–15) to the incidence in the baseline first 2 years (May 12, 2008, to May 11, 2010	Adjusted for observed increases in the number of children referred to clinicians per unit population over time by multiplying annual event counts by a correction factor that assumed the rate of referral in the absence of bias was constant	Radiologically confirmed pneumonia (WHO-defined) Adjusted annual Incidence/ 1,000 person-years<br><br>2-11months: 21<br>12-23 months: 15.3<br>2-4 years: 5.2	Radiologically confirmed pneumonia (WHO-defined) Adjusted annual Incidence / 1,000 person-years<br><br>2-11 months:<br>2014-2015: 16.2<br><br>12-23 months:<br>2014-2015: 10.9<br><br>2-4 years:<br>2014-2015: 4.1	2-11months	23 (7-36)	0.77 (0·64–0·93)
						12-23 months	29 (12-42)	0·71 (0·58–0·88)
						2-4 years	22 (1-39)	0·78 (0·61–0·99)
Ruvinsky 2018 [24] [51]	Argentina	Crude incidence rate ratios (IRR) were calculated between the pre-PCV period and the post-PCV period	None	Radiologically confirmed pneumonia (WHO-defined) Annual incidence rate /100 000 individuals annually	Radiologically confirmed pneumonia (WHO-defined) Annual incidence rate /100 000 individuals annually	0-11 months	53.4, $p < 0.05$	
						12-23 months	70.0, $p < 0.05$	
						24-59 months	73.3, $p < 0.05$	
						<5 years	64.9, $p < 0.05$	

[24] IRR calculated incorrectly and not appropriate to report

				0-11 months: 1,469.4<br><br>12-23 months: 1,075.6<br><br>24-59 months: 572.4<br><br><5 years (total): 847.5	0-11 months: 684.3<br><br>12-23 months: 322.5<br><br>24-59 months: 152.6<br><br><5 years (total): 296.9			
Take uchi 2020 **[52]**	Japan	Poisson regression was used to estimate incidence rates, incidence rate ratios and confidence intervals of CAP and PP.	None	Annual hospitalised of radiologically confirmed pneumonia (called CAP in the study) incidence per 1000 children:<br><5 years<br>2008 = 17.7<br>2012 = 14.3<br><br>5-15 years<br>2008 = 1.18<br>2012 = 2.64	Annual hospitalised of radiologically confirmed pneumonia (called CAP in the study) incidence per 1000 children;<br><5 years<br>2016 = 10.1<br>2017 = 10.6<br>2018 = 9.7<br><br>5-15 years<br>2016 = 1.41<br>2017 = 0.84	<5 years	45, p <0.001	0.55 (0.48–0.62
						5-15 years	41, p <0.001	0.59, (0.43–0.80)

					2018 = 0.69			

Table S7. Summary of results for Pneumococcal Pneumonia

Author	Country	Analytical Method	Adjustment factors	Incidence Rates		Vaccine Impact Results		
				Pre-PCV	Post-PCV	Age Group	Percent Reduction (%)(95% CI), p-value (if provided)	IRR (95% CI) (if provided)
Ben-Shimol 2018 [55]	Israel	Mean incidences during PCV13 periods and PCV7 periods were compared with Pre-PCV period. Crude IRRs and 95% CI comparing the post-PCV period to the pre-PCV period were calculated	None	Incidence (per 100,000 children) for pneumococcal pneumonia hospitalization<br><br><12 months: 17.7 (+/- 6.1)<br><br>12-23 months: 30.3 (+/-9.0)<br><br>24-59 months: 9.9 (+/- 3.1)	Incidence (per 100,000 children) for pneumococcal pneumonia hospitalization<br><br><12 months: 8.5<br><br>12-23 months: 10.0<br><br>24-59 months: 2.2	<12 months	52	0.48 (0.33-0.70)
						12-23 months	67	0.33(0.23-0.47)
						24-59 months	77	0.23 (0.15-0.35)
						<5 years	67	

Hammitt 2019 [56]	Kenya	Crude and adjusted IRRs and 95% CI comparing the post-PCV period to the pre-PCV period were calculated	Adjusted for confounding factors significant in the age specific all type IPD models: year (age groups < 2 months and <5 years), blood culture collection (age groups 5-14years). Year of vaccine introduction was excluded from analysis of PCV10 impact	Incidence pneumococcal pneumonia per 100,000 (95%CI)<br><br><5 years: 43.1(37.5-49.3)<br><br>5-14years: 7.1(5.4-9.2)	Incidence pneumococcal pneumonia per 100,000 (95%CI)<br><br><5 years: 8.6(5.2-13.4)<br><br>5-14years: 2.8(1.4-5.0)	<5 years	85 (66-93)	0.15(0.07-0.34)
						5-14 years	51, non-significant	0.49(0.21-1.11)
Meder 2020 [57]	Australia	Incidence rate ratios (IRRs) were calculated against the pre–universal period as baseline for both early PCV7 and post-	None	Incidence Rate (per 100,000 population)	Incidence Rate (per 100,000 population)	< 1 year	66	0.34 (.25–.45)
						1-4 years	50	0.50 (.43–.57)
						<5 years	45	

		PCV13 and against the pre-PCV13 period as baseline (pre-universal and pCV7 period) for the post-PCV13 period.		< 1 year = 18.6<br>1-4 years = 15.9	< 1 year = 6.2<br>1-4 years = 7.9			
Mackenzie 2017 [41]	The Gambia	Poisson distribution to calculate incidence rate ratios (IRRs) and 95% CIs of the incidence in the last 2 years of surveillance (2014–15) to the incidence in the baseline first 2 years (May 12, 2008, to May 11, 2010	adjusted for observed increases in the number of children referred to clinicians per unit population over time by multiplying annual event counts by a correction factor that assumed the rate of referral in the absence of bias was constant	Adjusted annual Incidence pneumococcal pneumonia 1,000 person-years<br>2-11months: 2.9<br>12-23 months: 2.6<br>2-4 years: 0.9	Adjusted annual Incidence pneumococcal pneumonia 1,000 person-years<br>2-11months: 1.2<br>12-23 months: 0.7<br>2-4 years: 0.3	2-11 months	58	Pneumococcal pneumonia (radiological): 0·31 (0·12–0·79)<br>Pneumococcal pneumonia (clinical): 0·42 (0·23–0·78)
						12-23 months	75	Pneumococcal pneumonia (radiological): 0·14 (0·05–0·35)<br>Pneumococcal pneumonia (clinical): 0·25 (0·12–0·53)
						2-4 years	64	Pneumococcal pneumonia

								(radiological): 0·34 (0·16–0·72)<br>Pneumococcal pneumonia (clinical): 0·43 (0·24–0·78)
Palmu 2017<br>[30]	Finland	Pneumonia rates in the target cohort were compared to rates in the combined reference cohorts using Poisson regression models. Relative rate reduction (percent) was calculated as (1 ±relative risk) 100%.	To reduce the effect seasonal variation on annual estimates yearly rates were calculated by epidemic years (July to June).	Incidence rate (per 1,000 person-years) Pneumococcal Pneumonia<br>Direct Target Cohort<br>Pre-PCV1 = 0.25<br>Indirect Target Cohort<br>Pre-PCV2= 0.18	Incidence rate per 1,000 person-years) Pneumococcal Pneumonia<br>Direct Target Cohort<br>Post-PCV1 = 0.06<br>Indirect Target Cohort<br>Post-PCV2 = 0.05	3-24 months	77 (64-86)	
						7-71 months	70 (49- 84)	
Takeuchi 2020<br>[52]	Japan	Poisson regression was used to estimate incidence rates, incidence	None	Incidence specific for pneumococcal pneumonia not		Authors report a decline, but do not provide a point estimate		

		rate ratios and confidence intervals of CAP and PP.		stated, see Appendix 6 for total CAP incidence rates			
Thorrington 2018[25] [35]	England	Composite Control Method	To account for biases arising from potential secular trends in admission practice over the study period, the IRRs of each disease endpoint were compared to the IRRs of five control conditions that should not be affected by changes in the introduction of	Not stated[26]	< 2 years	81	0.19 [0.15, 0.25]
					2-4 years	53	0.47 [0.36, 0.65]
					5-14 years	69	0.31 [0.27, 0.41]

[25] Adjusted IRR (rIRR) are calculated with [min-max] not 95% CI

[26] Only provides incidence rate values on graphs, does not provide incidence rate values

			the PCV programme				

Table S8. Summary of results for Pneumonia Mortality								
**Author**	**Country**	**Analytical Method**	**Adjustment factors**	**Incidence Rates or Case Fatality Ratio**		**Vaccine Impact Results**		
				**Pre-PCV**	**Post-PCV**	**Age Group**	**Percent Reduction (%)(95% CI), p-value (if provided)**	**IRR (95% CI) (if provided)**
Becker-Dreps 2017 [15]	Nicaragua	Adjusted IRR and 95% CI were estimated using GEE for vaccine period compared to pre-vaccine period	Controlled for municipality to account for distance to patients home municipality to the hospital and potential differences in care seeking and immunization coverage by municipality category	Incidence Rate of Pneumonia related deaths (1,000 person-years)<br><br>< 12months Pre-PCV: 93.9<br>12-23 months Pre-PCV: 4.1<br>25-59 months re-PCV: 5.7	Incidence Rate of Pneumonia related deaths (1,000 person-years)<br><br>< 12months Post-PCV: 37 (96.4)<br>12-23 months Post-PCV: 7.7<br>25-59months Post-PCV: 5.1	0-4 years	N/A, non-significant	1.01 (0.67,1.52)
						5-14 years	78, non-significant	0.22 (0.02,2.47)

				5-14 years Pre: 1.2	5-14 years Post-PCV: 0.3			
Schuck-Paim 2019[27]<br>[58]	Brazil	Two complementary regression methods (synthetic control analysis and time-trend adjustment) were used to assess changes in pneumonia deaths after the introduction<br>of PCV10. In both methods, the models were fit to data from the pre-vaccine period alone and controlled for seasonal variations with quarterly dummy variables. Rate ratios were calculated by comparing observed and counterfactual rates in the post-PCV10	Adjusted for seasonality and hospital use<br>The<br>2009 influenza virus H1N1 pandemic affected Brazil from<br>April, 2009, to March, 2010; therefore, this period was<br>excluded from all analyses.	Pneumonia mortality (per 100 000 population) <5 years<br><br>1980: 147.0<br>2010: 16.1	Annual Pneumonia mortality (per 100 000 population) in 2010<br><br>3-11months: 37<br>3-23 months: 24<br>3-59months: 11	3-11 months	12 (–6 to 12)	Synthetic Control Method: 0.88(.78, 1.06)<br>Time Trends Method: 0.90(.82, .99)
						3-23 months	12 (–6 to 13)	Synthetic Control Method: 0.88(.77, 1.06)<br>Time Trends Method: 0.90(.82, .98)
						3-59 months	8 (–9 to 19)	Synthetic Control Method: 0.92(.81, 1.09)<br>Time Trends Method: : 0.95(.87, 1.03)
						<5 years	10	

[27] Rate Ratios calculated with 95% CrI instead of 95% CI

		period using a Bayesian setting that allowed us to quantify uncertainty associated with variable selection and variation in the data				*All estimates not significant<br>Background rate of pneumonia mortality:<br>Decreasing before PCV introduction, ’% decrease 1980-2010 = 90%)		
Kabore 2020<br>[39]	Burkina Faso	Pre and Post PCV trends were generated with segmented regression analysis. Calculated IRR and incidence rates	Adjusted for seasonality, pre-PCV trends and a binary variable for a free care policy implemented nationwide in 2017	Not stated	Not stated	0-59 months	Change in intercept: 51, p= 0.002<br>Change in slope: p= 0.004	Change in intercept: 0.49 (0.31; 0.78)<br>Change in slope: 0.965 (0.942; 0.989)
						0-23 months	Change in intercept: 40, p= 0.05, non-significant<br>Change in slope: 5, p <0.001	Change in intercept: 0.60 (0.37; 0.99)<br>Change in slope: 0.95 (0.920; 0.971)
						24-59 months	Change in intercept: 78, p= 0.003<br>Change in slope: N/A, p=	Change in intercept: 0.22 (0.08; 0.60)<br>Change in slope: 1.031 (0.980; 1.083)

							0.24, non-significant	
Vestjens 2019[28] [43]	The Netherlands	Differences in incidences and proportions between the time periods were tested with v2 tests and RRs with 95% CI were calculated. (No further details are stated)	If considered appropriate, multivariable logistic regression analyses were performed to adjust for age and/or comorbidities when assessing associations between time periods and outcomes. (No further details stated)	Case Fatality Ratio[29] <5 years: 5.6 5-17 years: 4.4	Case Fatality Ratio[30] <5 years: 8.1 5-17 years: 4.	<5 years	N/A, non-significant	1.46 (0.37–5.80)
						5-17 years	22, non-significant	0.88 (0.06–13.3)

[28] Uses the term “relative risk” in result calculation, but appears to be equal to a rate ratio calculation

[29] Case Fatality Ratios are not specific for pneumonia mortality but include: meningitis, pneumonia, pneumonia with empyema, bacteraemia without a focus and bacteraemia without a focus

[30] Case Fatality Ratios are not specific for pneumonia mortality but include: meningitis, pneumonia, pneumonia with empyema, bacteraemia without a focus and bacteraemia without a focus

Naucler 2019 [29]	Sweden	Poisson regression was used to calculate incidence rate ratios (IRR) with 95% confidence intervals (CI to compare pneumonia in 2014–15 (post-vaccination period) with 2005–06 (pre-vaccination period).	Adjusted for trends in admittance practices using four control conditions. Age adjusted incidence rates to year 2005	Incidence of 30 day all-cause pneumonia mortality<br>Pre-PCV per 100,000 person-years:<br><br>< 2 years: 1.3<br>2-4 years: 0.7<br>5-17 years: 0.3	Incidence of 30 day all-cause pneumonia mortality<br>Post-PCV per 100,000 person-years:<br><br>< 2 years: 0.5<br>2-4 years: 0.3<br>5-17 years: 0.3	< 2 years	64, non-significant	0.36 (0.07–1.83)
						2-4 years	58, non-significant	0.42 (0.08–2.31)
						5-17 years	10, non-significant	0.9 (0.35–2.33)
				Incidence of 30 day PL-pneumonia mortality<br>Pre-PCV per 100,000 person-years:<br><br>< 2 years: 0.0<br>2-4 years: 0.2<br>5-17 years: 0.04	Incidence of 30 day PL-pneumonia mortality<br>Post-PCV per 100,000 person-years:<br><br>< 2 years: 0.0<br>2-4 years: 0.0<br>5-17 years: 0.0	30 day PL-pneumonia mortality had too few cases to calculate an IRR		

Mpabalwani 2019 [28]	Zambia	Monthly case counts during the pre-PCV10 period were used in a negative binomial regression models to calculated predicted case counts had PCV not been introduced.<br><br>The decline in hospitalizations or deaths was calculated as the ratio of the observed to the expected data in the post-PCV10 period<br><br>Chi-square test was used to compare case fatality ratios between pre- and post-PCV10 periods by age group	Adjusted for seasonal pattern of the case counts and UTH pneumonia models, included monthly hospitalization counts for the control conditions and an interaction term between the control hospitalization counts and the continuous time indicator.	In-hospital case-fatality ratio for pneumonia (MOH Data)<br><br>Children < 1 year: 6.5%<br><br>Children 1-4 years: 3.5%<br><br>In-hospital case-fatality ratio for pneumonia (UTH Data)<br><br>Children < 1 year: 15.3%<br><br>Children 1-4 years: 8.8%	In-hospital case-fatality ratio for pneumonia (MOH Data)<br><br>Children < 1 year: 5.9%<br><br>Children 1-4 years: 3.1%<br><br>In-hospital case-fatality ratio for pneumonia (UTH Data)<br><br>Children < 1 year: 16.3%<br><br>Children 1-4 years: 15.2%	< 1 year	no significant changes in the number of in-hospital pneumonia deaths
						< 4 years	no significant changes in the number of in-hospital pneumonia deaths

Congdon 2020 [19]	Botswana	A seasonally adjusted interrupted time-series analysis using negative binomial regression model was used to evaluate the effect of introduction of these vaccines on child pneumonia hospitalizations and deaths.	Adjusted for seasonal trends	Annual Rate of Change (95% CI) in the Number of Pneumonia related deaths<br><br>All ages:<br>Rate: 1.59 (CI: 0.87-2.90), increasing by 59%<br>1-11months:<br>Rate: 1.44 (CI: 0.70,2.99)<br>12-59months:<br>Rate: 1.94 (CI: 0.82, 4.61)	Annual Rate of Change (95% CI) in the Number of Pneumonia related deaths<br><br>All ages:<br>Rate: 0.78 (CI: 0.67,0.92)<br>1-11months:<br>Rate: 0.82 (CI: 0.69,1.01)<br>12-59months:<br>Rate: 0.70 (CI: 0.54, 0.90)	1-59 months	% Decline of 22 per year during the post-vaccine period	Annual rate of decline in post-PCV period: 0.78 (0.67- 0.92)
Camargos 2020 [18]	Brazil	Trend estimates and annual percent change values were estimated - using joint point regression software and Monte Carlo permutation	None	LRI - Mortality Incidence rate (x 100,000)<br><br>1990-1995: -6.5(-6.8;-6.2)	LRI - Mortality Incidence rate (x 100,000)<br><br>2010-2017: -6.5(-6.7;-6.3)	< 5 years	Annual % change decline in post-PCV period: 6.5 (6.3-6.7), not statistically significant	

				1995-2000: -7.3(-7.7;-6.8)<br>2000-2003: -8.3(-9.7;-7.0)<br>2003-2010: -7.0(-7.2;-6.7)<br>Annual change % LRI mortality rate<br>1990-2009<br>1991: -7.3<br>1992: -6.4<br>1993: -5.9<br>1994: -6.4<br>1995: -6.4<br>1996: -7.9<br>1997: -7.4<br>1998: -7.1<br>1999: -6.4<br>2000: -7.8<br>2001: -8.8<br>2002: -8.4	Annual change % lri mortality rate<br>2010: -7.6<br>2011: -6.1<br>2012: -6.1<br>2013: -5.8<br>2014: -7.1<br>2015: -7.8<br>2016: -5.9<br>2017: -6.2			

				2003: -7.7<br>2004: -6.9<br>2005: -7.0<br>2006: -7.2<br>2007: -7.2<br>2008: -7.1<br>2009: -6.4				
Jimenez-Trujillo 2017<br>[25]	Spain	Incidence rates per 100,000 inhabitants calculated: no. of admissions per year / no. of people in that population group according to census data. Linear joint point regression to identify trend changes in CAP incidence rates, then a time-trend analysis was performed using Poisson regression	Poisson regression, adjusted by age and sex when needed, and the year 2009was analysed separately - year of H1N1 influenza pandemic	Pneumonia Related In-Hospital Mortality (% of CAP admissions) all ages < 18 years<br>2001-2003: 4.1%	Pneumonia Related In-Hospital Mortality (% of CAP admissions) all ages < 18 years<br>2012-2014: 2.8%	IHM following CAP fell significantly in children and adolescents from the pre-PCV to the post-PCV period: Odds Ratio 0.82 (95%CI 0.77–0.89)[31]		

[31] Reporting an odds ratio not a Rate Ratio

Sgambatti 2016 [33]	Brazil	Linear regression using Poisson distribution to assess the rate change in the post-PCV period compared to the pre-PCV period	Adjusted for secular trends	Pneumonia related Case Fatality Ratio<br><br>Clinical Pneumonia: 18<br>X-Ray confirmed pneumonia: 7	Pneumonia related Case Fatality ratio<br><br>Clinical Pneumonia: 19<br>X-Ray confirmed pneumonia: 13	Children aged 2-35 months, no significant changes in pneumonia related mortality for either clinical or X-ay confirmed pneumonia, p = 0.531 and p-= 0.100 respectively.

PRISMA 2020 Checklist

Section and Topic	Item #	Checklist item	Location where item is reported
**TITLE**			
Title	1	Identify the report as a systematic review.	Line 1
**ABSTRACT**			
Abstract	2	See the PRISMA 2020 for Abstracts checklist.	
**INTRODUCTION**			
Rationale	3	Describe the rationale for the review in the context of existing knowledge.	Line 99-101
Objectives	4	Provide an explicit statement of the objective(s) or question(s) the review addresses.	Line 102-107
**METHODS**			
Eligibility criteria	5	Specify the inclusion and exclusion criteria for the review and how studies were grouped for the syntheses.	Line 120-136
Information sources	6	Specify all databases, registers, websites, organisations, reference lists and other sources searched or consulted to identify studies. Specify the date when each source was last searched or consulted.	Line 110-113
Search strategy	7	Present the full search strategies for all databases, registers and websites, including any filters and limits used.	Supplementary material
Selection process	8	Specify the methods used to decide whether a study met the inclusion criteria of the review, including how many reviewers screened each record and each report retrieved, whether they worked independently, and if applicable, details of automation tools used in the process.	Line 114-118
Data collection process	9	Specify the methods used to collect data from reports, including how many reviewers collected data from each report, whether they worked independently, any processes for obtaining or confirming data from study investigators, and if applicable, details of automation tools used in the process.	Line 114-118
Data items	10a	List and define all outcomes for which data were sought. Specify whether all results that were compatible with each outcome domain in each study were sought (e.g. for all measures, time points, analyses), and if not, the methods used to decide which results to collect.	Line 128-129
	10b	List and define all other variables for which data were sought (e.g. participant and intervention characteristics, funding sources). Describe any assumptions made about any missing or unclear information.	Line 126-136
Study risk of bias assessment	11	Specify the methods used to assess risk of bias in the included studies, including details of the tool(s) used, how many reviewers assessed each study and whether they worked independently, and if applicable, details of automation tools used in the process.	Line 139-146
Effect measures	12	Specify for each outcome the effect measure(s) (e.g. risk ratio, mean difference) used in the synthesis or presentation of results.	Line 127
Synthesis methods	13a	Describe the processes used to decide which studies were eligible for each synthesis (e.g. tabulating the study intervention characteristics and comparing against the planned groups for each synthesis (item #5)).	Line 126-138
	13b	Describe any methods required to prepare the data for presentation or synthesis, such as handling of missing summary statistics, or data conversions.	N/A
	13c	Describe any methods used to tabulate or visually display results of individual studies and syntheses.	Line 145-146
	13d	Describe any methods used to synthesize results and provide a rationale for the choice(s). If meta-analysis was performed, describe the model(s), method(s) to identify the presence and extent of statistical heterogeneity, and software package(s) used.	Line 145-146
	13e	Describe any methods used to explore possible causes of heterogeneity among study results (e.g. subgroup analysis, meta-regression).	Line 146-148
	13f	Describe any sensitivity analyses conducted to assess robustness of the synthesized results.	N/A
Reporting bias assessment	14	Describe any methods used to assess risk of bias due to missing results in a synthesis (arising from reporting biases).	Line 139-144
Certainty	15	Describe any methods used to assess certainty (or confidence) in the body of evidence for an outcome.	N/A

PRISMA 2020 Checklist

Section and Topic	Item #	Checklist item	Location where item is reported
assessment			
**RESULTS**			
Study selection	16a	Describe the results of the search and selection process, from the number of records identified in the search to the number of studies included in the review, ideally using a flow diagram.	Figure 1
	16b	Cite studies that might appear to meet the inclusion criteria, but which were excluded, and explain why they were excluded.	Figure 1
Study characteristics	17	Cite each included study and present its characteristics.	Table 1
Risk of bias in studies	18	Present assessments of risk of bias for each included study.	Supplementary material
Results of individual studies	19	For all outcomes, present, for each study: (a) summary statistics for each group (where appropriate) and (b) an effect estimate and its precision (e.g. confidence/credible interval), ideally using structured tables or plots.	Table 2
Results of syntheses	20a	For each synthesis, briefly summarise the characteristics and risk of bias among contributing studies.	N/A. Synthesis not conducted
	20b	Present results of all statistical syntheses conducted. If meta-analysis was done, present for each the summary estimate and its precision (e.g. confidence/credible interval) and measures of statistical heterogeneity. If comparing groups, describe the direction of the effect.	N/A
	20c	Present results of all investigations of possible causes of heterogeneity among study results.	N/A
	20d	Present results of all sensitivity analyses conducted to assess the robustness of the synthesized results.	N/A
Reporting biases	21	Present assessments of risk of bias due to missing results (arising from reporting biases) for each synthesis assessed.	N/A. Synthesis not conducted
Certainty of evidence	22	Present assessments of certainty (or confidence) in the body of evidence for each outcome assessed.	Confidence intervals included in Table 2
**DISCUSSION**			
Discussion	23a	Provide a general interpretation of the results in the context of other evidence.	Line 290-300
	23b	Discuss any limitations of the evidence included in the review.	Line 352-365
	23c	Discuss any limitations of the review processes used.	Line 352-365
	23d	Discuss implications of the results for practice, policy, and future research.	Line 343-350
**OTHER INFORMATION**			
Registration and protocol	24a	Provide registration information for the review, including register name and registration number, or state that the review was not registered.	Not registered
	24b	Indicate where the review protocol can be accessed, or state that a protocol was not prepared.	Line 135
	24c	Describe and explain any amendments to information provided at registration or in the protocol.	None
Support	25	Describe sources of financial or non-financial support for the review, and the role of the funders or sponsors in the review.	Line 379-382
Competing interests	26	Declare any competing interests of review authors.	Line 378
Availability of data, code and	27	Report which of the following are publicly available and where they can be found: template data collection forms; data extracted from	Available upon

PRISMA 2020 Checklist

Section and Topic	Item #	Checklist item	Location where item is reported
other materials		included studies; data used for all analyses; analytic code; any other materials used in the review.	request

From: Page MJ, McKenzie JE, Bossuyt PM, Boutron I, Hoffmann TC, Mulrow CD, et al. The PRISMA 2020 statement: an updated guideline for reporting systematic reviews. BMJ 2021;372:n71. doi: 10.1136/bmj.n71

For more information, visit: http://www.prisma-statement.org/
